# Evolution of *Salmonella enterica* serotype Typhimurium driven by anthropogenic selection and niche adaptation

**DOI:** 10.1371/journal.pgen.1008850

**Published:** 2020-06-08

**Authors:** Matt Bawn, Nabil-Fareed Alikhan, Gaëtan Thilliez, Mark Kirkwood, Nicole E. Wheeler, Liljana Petrovska, Timothy J. Dallman, Evelien M. Adriaenssens, Neil Hall, Robert A. Kingsley

**Affiliations:** 1 Quadram Institute Biosciences, Norwich Research Park, Norwich, United Kingdom; 2 Earlham Institute, Norwich Research Park, Norwich, United Kingdom; 3 Centre for Genomic Pathogen Surveillance, Wellcome Sanger Institute, Cambridge, United Kingdom; 4 Animal and Plant Health Agency, Addlestone, United Kingdom; 5 Gastrointestinal Bacteria Reference Unit, National Infection Service, Public Health England, London, United Kingdom; 6 University of East Anglia, Norwich, United Kingdom; University of Warwick, UNITED KINGDOM

## Abstract

*Salmonella enterica* serotype Typhimurium (*S*. Typhimurium) is a leading cause of gastroenteritis and bacteraemia worldwide, and a model organism for the study of host-pathogen interactions. Two *S*. Typhimurium strains (SL1344 and ATCC14028) are widely used to study host-pathogen interactions, yet genotypic variation results in strains with diverse host range, pathogenicity and risk to food safety. The population structure of diverse strains of *S*. Typhimurium revealed a major phylogroup of predominantly sequence type 19 (ST19) and a minor phylogroup of ST36. The major phylogroup had a population structure with two high order clades (α and β) and multiple subclades on extended internal branches, that exhibited distinct signatures of host adaptation and anthropogenic selection. Clade α contained a number of subclades composed of strains from well characterized epidemics in domesticated animals, while clade β contained multiple subclades associated with wild avian species. The contrasting epidemiology of strains in clade α and β was reflected by the distinct distribution of antimicrobial resistance (AMR) genes, accumulation of hypothetically disrupted coding sequences (HDCS), and signatures of functional diversification. These observations were consistent with elevated anthropogenic selection of clade α lineages from adaptation to circulation in populations of domesticated livestock, and the predisposition of clade β lineages to undergo adaptation to an invasive lifestyle by a process of convergent evolution with of host adapted *Salmonella* serotypes. Gene flux was predominantly driven by acquisition and recombination of prophage and associated cargo genes, with only occasional loss of these elements. The acquisition of large chromosomally-encoded genetic islands was limited, but notably, a feature of two recent pandemic clones (DT104 and monophasic *S*. Typhimurium ST34) of clade α (SGI-1 and SGI-4).

## Author summary

*Salmonella* Typhimurium is a leading cause of foodborne illness worldwide. Our current understanding of the biology of *Salmonella* is largely based on studies using just two laboratory strains of *S*. Typhimurium, with similar characteristics. Yet this pathogen exhibits a remarkable diversity in host range, outcome of infection, and risk to human health. To investigate the genetic basis of this diversity, we have explored the genetic relationship of a collection of isolates that represent a substantial portion of the diversity of *S*. Typhimurium, using whole genome sequencing. *S*. Typhimurium evolved forming two major groups that differ in their distribution in livestock and wild avian species. The livestock-associated group contained isolates commonly affecting human health and were often drug resistant, while the wild avian-associated group was rarely associated with drug resistance and less frequently associated with human infection. We report distinct evolutionary processes acting on subgroups of *S*. Typhimurium including loss of information content of genomes, and gain or loss of genes predicted to affect functions such as antimicrobial resistance, disease potential, and environmental survival, and functional diversification. This study provides a framework in which to understand the epidemiology of *Salmonella* and improve assessment of its risk to animal and human health.

## Introduction

Bacteria of the genus *Salmonella* are a common cause of foodborne disease. Most of the approximately 2500 serovars cause gastroenteritis in humans and other animals, while some have evolved host adaptation associated with extra intestinal disseminated infections in specific host species [1]. For example, *Salmonella enterica* serovar Typhimurium (*S*. Typhimurium) and *S*. Enteritidis circulate in multiple vertebrate host species and cause food borne infections in the human population. These and other non-typhoidal Salmonella serotypes result in an estimated 75 million cases and 27 thousand deaths from gastroenteritis worldwide [2]. *S*. Typhi and *S*. Paratyphi A circulate exclusively in the human population and cause an estimated 2.5 million infections resulting in 65 thousand deaths each year as a result of the disseminated disease typhoid and paratyphoid disease [2]. Similarly, other serotypes evolved host adaptation to specific non-human host species, such as *S*. Gallinarum with poultry, *S*. Dublin with cattle, and *S*. Choleraesuis with pigs, where they are associated with disseminated infections [1].

Although *S*. Typhimurium is considered to be a broad host range serotype, the epidemiological record of *S*. Typhimurium phage types identified several *S*. Typhimurium pathovariants with distinct host range, pathogenicity and risk to food safety [3, 4]. The pathovariant commonly associated with this serotype, has a broad host range and is associated with gastroenteritis in the human population. Such broad host range strains of *S*. Typhimurium account for the majority of those isolated by public health surveillance in England, presumably because they are common in many species of livestock and poultry, the primary zoonotic reservoir for human infections [5]. The epidemiological record of this pathovariant is characterised by successive waves of dominant clones identified historically by their phage type, that account for up to 60% of all human infections for several years, before being replaced by a subsequent strains [6]. Dominant clonal groups have been characterized by strains of phage types DT9, DT204/49 complex, DT104, and the current monophasic *S*. Typhimurium (*S*. 4,[5],12:i:-) sequence type 34 (ST34), since around the middle of the last century [7–10]. In contrast, some phage types are common in clonal groups typically associated with a restricted host range, and in some cases altered pathogenicity. For example, clonal groups of *S*. Typhimurium DT8, DT2 and DT56 circulate in populations of ducks, pigeon, and passerine birds, respectively, and only rarely cause gastroenteritis in the human population [11–13]. Also, specific clonal groups of *S*. Typhimurium ST313 are associated with disseminated disease (invasive non-typhoidal *Salmonella*, iNTS) in sub-Saharan Africa [14, 15].

In this study we report the population structure, gene flux, recombination and signatures of functional diversification in the whole genome sequence of 131 strains of *S*. Typhimurium with well characterised epidemiology. To assist in our analysis, we also report high quality complete and closed whole genome sequence of six additional reference genomes, representing diversity within the population structure not represented by previously reported sequence data.

## Results

### Population structure of *S*. Typhimurium consists of two high-order clades containing strains with distinct epidemiology

**Fig 1:**
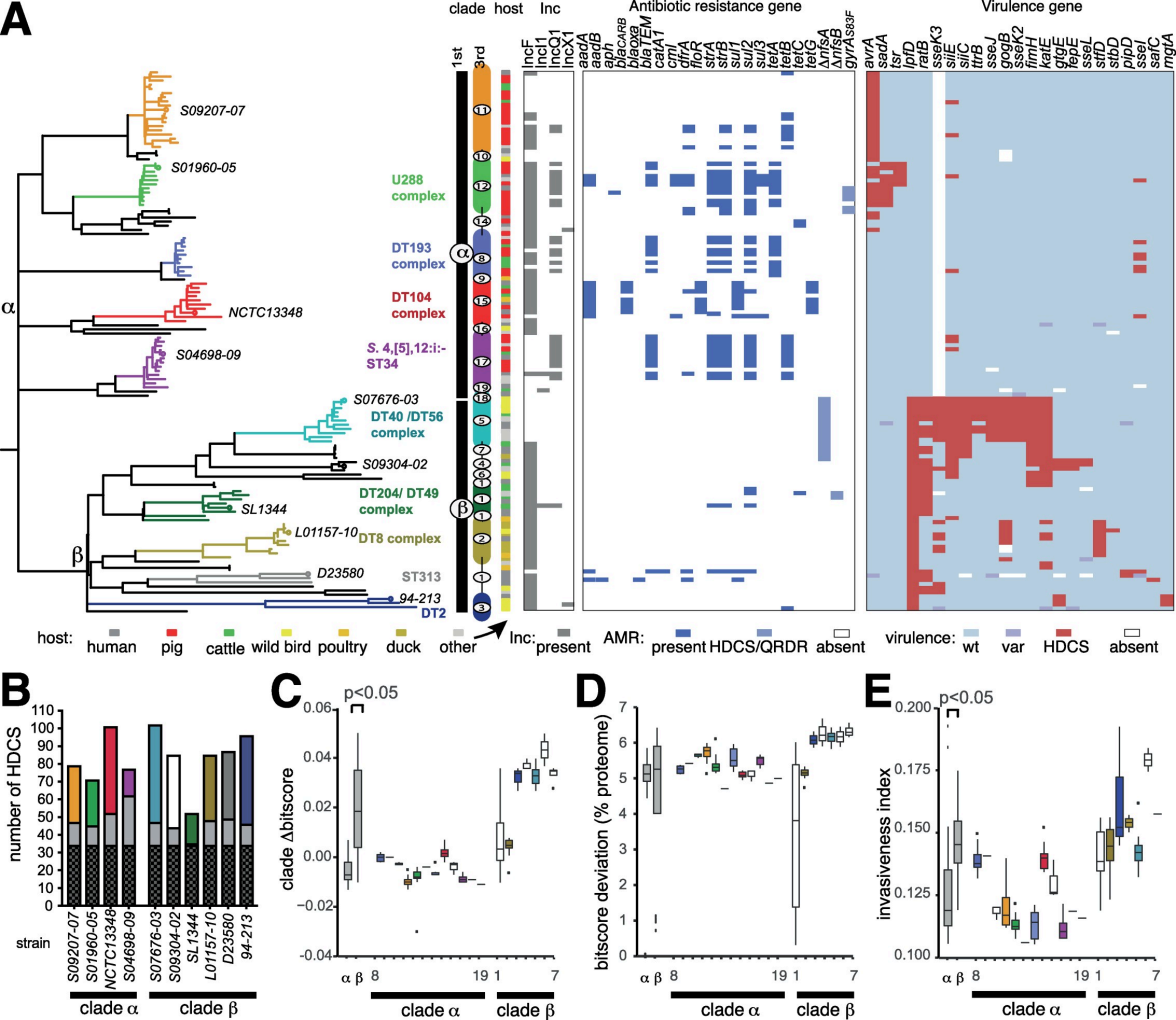
Phylogenetic relationship of the ST19 *Salmonella* Typhimurium phylogroup. (A) Maximum likelihood phylogenetic tree and based on sequence variation (SNPs) in the core genome with reference to *S*. Typhimurium strain SL1344. The root was identified using *S*. Heidelberg (accession number NC_011083.1) as the outgroup. 1^st^ (α and β) and 3^rd^ (α11–19 and β1–7) are indicated (vertical bars). Phage type complexes associated with the third-level clusters are indicated (bold type) colour coded with the lineages and representative strains from third level clusters (italicized type). The source of each isolate in the tree is indicated by filled boxes colour coded as indicated in the inset key (arrow). The presence of replicon sequence (grey box), antimicrobial resistance genes (blue box) and hypothetically disrupted coding sequence (HDCS) of virulence related genes (red box) in short read sequence data are indicated. HDCS in *nfsA* and *nfsB* resulting in resistance to nitrofuran antibiotics and ns SNPs resulting in substitutions in the QRDR of GyrA are indicated (light blue boxes). (B) Bars indicate the number of ancestral (black), phage or insertion sequence elements (grey), chromosomal gene (colour coded with lineages in Fig 1A) HDCS in the genome of representative strains from each third level clade. (C) Box plots indicate the mean Δbitscore (DBS: bitscore SL1344 –test strain bitscore) of proteomes in third level clades. (D) Box plot indicates the percentage of the proteome of the proteome of isolates from each third level clade with a non-zero bitscore (bitscore SL1344 –test strain bitscore >0 or <0) as an estimate of function divergence. (E) Box plots indicate the mean invasiveness index per genome, the fraction of random forest decision trees voting for an invasiveness phenotype based on training on the DBS of a subset of the proteome of ten gastrointestinal and extraintestinal pathovar serotypes.

Variant sites (38739 SNPs) in the core genome sequence of 134 *S*. Typhimurium strains representing commonly isolated phage types revealed two diverse phylogroups composed of three strains of ST36 that clustered separately from the remaining 131 Typhimurium isolates (S1 Fig). The two phylogroups were more similar to one another than any other serotype, including a closely related isolate of serotype *S*. Heidelberg. Since the majority of *S*. Typhimurium formed a large number of relatively tightly clustered isolates, predominantly of ST19, we focussed on the analysis of the population structure and evolution of this phylogroup. A phylogenetic tree constructed using variant sites (8382 SNPs) in the core genome sequence of the 131 *S*. Typhimurium strains and rooted with *S*. Heidelberg, revealed a ‘star’ topology with relatively long internal branches extending from a hypothetical common ancestor, and diversification at the terminal branches (Fig 1).

The population structure determined using a three-level hierarchical Bayesian approach [16] resolved *S*. Typhimurium into two major clades, designated α and β, seven second level clades and 18 third-level clades (α 8–18 and β 1–7) (S1 Table). In many cases, third level clades corresponded to known epidemic clades, with the exception of β1 that was a poorly defined basal clade, and we therefore focused on 1^st^ and 3^rd^ level clades. Clade β was defined by an internal branch that originated from a common ancestor of the basal clade α, defined by approximately 100 core genome SNPs.

Despite relatively few SNPs distinguishing clade α and β, these clades exhibited distinct epidemiology characterised by association predominantly with livestock, including cattle, pigs and poultry (clade α) or avian species, including wild species (clade β). Strikingly all pig isolates from our sampling were located in clade α. Cattle isolates were in both first order clades (11 in clade α 7 in clade β), but in clade β they had a relatively limited distribution with five of the isolates from a subclade containing the DT204/49 complex of strains associated with a cattle associated epidemic in the 1970’s [8]. Clade α contained strains from several previously described epidemics in livestock animal species, including in pigs (α12, U288)[17], two clades associated with recent pandemic clonal groups associated with pigs, cattle and poultry (α17, monophasic Typhimurium ST34 and α15, DT104) [18–20], and potentially epidemic clades not previously described in the literature, consisting of isolates from pigs, cattle and poultry (α8 and α11). Clade-β was characterised by many long internal branches, indicative of a relatively high level of root to tip sequence divergence, relative to those in Clade-α. In contrast, clade-β contained several third-level clades previously described as host-adapted, particularly for avian species such as passerine birds (β5, DT56), duck (β2, DT8) and pigeon (β3 DT2) [21], and ST313 that includes two sub-clades specifically associated with disseminated disease in sub-Saharan Africa [22].

To estimate the coverage of clinical isolates from the UK and global *S*. Typhimurium by our dataset, we compared the 131 *S*. Typhimurium genomes with 1697 *S*. Typhimurium genomes isolated from human clinical infection in the UK in 2014 and 2015 described previously [23], and 14,478 genomes from non-UK isolates, excluding ST36, in the EnteroBase database [24] (S2 Fig). With both datasets, we grouped these genomes according to their cgMLST Hierarchical Clusters at the level of 100 alleles difference (HierCC HC100) as defined in EnteroBase [24]. This provided an estimation of the genomic diversity within *S*. Typhimurium. We then determined the proportion of HierCC groups that contained a genome from our dataset, and the number of genomes in shared clusters. In the first comparison, 38.5% of hierarchical clusters were represented in our dataset and UK clinical isolates. Shared HC’s contained 95.1% of genomes from clinical infections because most of the unrepresented HC’s contained few genomes. In regard to the rest of the world, 17% of HC’s were represented in our dataset, but shared HierCCs accounted for 71.5% of the EnteroBase genomes. Therefore, although our dataset represents less than half of the known hierarchical clusters, it does cover the majority of genomes in available databases.

### Antimicrobial resistance genes and plasmid replicons are common in isolates associated with livestock

The presence of multiple third-level clades associated with recent livestock associated epidemic strains in the first level clade α and the relative paucity in clade β suggested that they may be under differential anthropogenic selection pressure. A key anthropogenic selection pressure on microbial populations circulating in livestock is the widespread use of antimicrobial drugs in animal husbandry. Consistent with their distinct epidemiology, antimicrobial resistance (AMR) genes were common in clades α (mean of 2.7 per strain) and relatively rare in β (mean 0.38 per strain) (Fig 1A and S2 Table). Indeed, AMR genes in clade-β were restricted to strains from DT204/49 complex known to be associated with cattle [8] and the ST313 associated with disseminated disease in sub-Saharan Africa commonly treated with antibiotics [22].

Resistance to fluoroquinolone and nitrofuran antimicrobials is associated with sequence polymorphisms in the *gyrA* and *parC*, and *nsfA* and *nsfB*, respectively [25, 26]. Of four substitutions in the quinolone resistance determining region (QRDR) of GyrA and ParC, just GyrA S83F was present in six isolates, four from α12 (pig-associated U288) and two from α8 (DT104 complex), had a sequence polymorphism resulting in GyrA S83F. No other mutations affecting the QRDR were detected. Nitrofuran resistance has been linked to HDCS due to insertion sequence (IS) elements or sequence polymorphisms, primarily of the *nsfA* gene, but occasional secondary mutations in *nsfB* [26]. The *nsfA* gene was present as an HDCS in just two isolates from subclade β1 and *nsfB* in 22 isolates from subclade β5 (DT56 complex).

AMR genes are commonly present on plasmids and we therefore determined the presence of plasmid replicon sequence in short read sequence data from the 131 strains in clades α and β. The IncQ1 plasmid replicon, previously associated with antibiotic resistance [27] was widespread, particularly in clade α. The IncF replicon corresponding to the presence of the virulence plasmid pSLT [28] was also widespread in *S*. Typhimurium as expected, and associated with antibiotic resistance in a number of subclades including ST313 and U288 complex [14, 29]. The pSLT plasmid varied in size ranging from 96 to 167kb (Table 1), and assembly of pSLT from short read sequence from all 131 isolates indicated no significant difference in the mean size between clades α and β. The pSLT plasmid was notably absent from a number of third-level clades including β5 (DT56), α11 and α17 (monophasic *S*. Typhimurium ST34).

**Table 1:** Characteristics of complete and closed whole genome sequence of *S*. Typhimurium reference strains.

strain	ST	PT^a^	accession no.	clade	chrom size^b^	pSLT^c^	P1^c^	P2^c^	ECC ^d^	CDS^e^	SNPs^f^	Prophage^g^	total HAC^h^	spec. HAC	year isolation	source
SO9207-07	19	DT170B	PRJEB34598	α11	4.92	-	-	-	0	4592	973	7 (5:1:1)	48	7	2007	Pig
SO1960-05	19	U288	PRJEB34597	α12	4.90	154.4	19.4	18.2	3	4832	1272	8 (5:2:1)	57	16	2005	Pig
NCTC13348	19	DT104	HF937208.1	α15	4.93	94.0	-	-	1	4751	1083	9 (5:3:1)	63	25	1988	Human
SO4698-09	34	DT193	LN999997.1	α17	5.04	-	-	-	0	4754	1010	9 (6:2:1)	43	7	2009	Cattle
SO7676-03	19	DT56	PRJEB34599	β5	4.88	-	-	-	0	4570	797	6 (4:1:1)	75	26	2003	Bird
SO9304-02	19	DT41	PRJEB34596	β4	5.05	117.4	32.2		3	4685	943	8 (5:2:1)	78	15	2002	Cattle
SL1344	19	DT44	FQ312003.1	β1	4.88	93.8	86.9	8.7	3	4771	0	9 (4:3:2)	42	2	~1966	Cattle
LO1157-10	19	DT8	PRJEB34595	β2	4.86	93.8	23.9	22.2	3	4706	1101	7 (4:2:1)	62	19	2010	Duck
D23580	313	-	FN424405.1	β1	4.88	117.0	84.6	-	2	4790	901	7 (5:2:0)	57	9	2004	Human
A130	313	DT56v	PRJEB34594	β1	4.93	166.9	-	-	1	4812	901	8 (5:2:1)	62	13	2001	Human
94–213	98	DT2	HG326213.1	β3	4.82	93.8	-	-	1	4598	903	5 (3:1:1)	66	20	1994	Pigeon

^a^ Phage type / Sequence type

^b^ Chromosome size (Mbp)

^c^ contig size (Kbp)

^d^ extra chromosomal contigs (ECC), putative plasmid sequences

^e^ Number of predicted coding sequences

^f^ number of SNPs with reference to SL1344

^g^ Number of prophage (intact, incomplete, candidate)

^h^ total number of pseudogenes with reference to SL1344 allele, ^i^ number of clade-specific pseudogenes (S1 Table), ^i^ mean delta bitscore (DBS) with reference to SL1344 protein orthologue.

Many key virulence genes of *Salmonella enterica* are present on *Salmonella* pathogenicity islands (SPIs) [30]. In general, SPI-1 to 6 were highly conserved, consistent with their key role in pathogenesis. However, SPI-4 containing the *sii* locus that encodes a giant adhesin secreted by a type I secretions system [31], exhibited elevated sequence divergence in several β subclades (S3 Fig). SPI-6 that encodes a type VI secretion system that mediates a cell contact dependent mechanisms of interbacterial antagonism involved in colonisation of the intestine [32], exhibited moderate sequence variation in isolates in subclade β7.

### Distinct patterns of genome degradation and signatures of functional divergence and invasiveness in clades α and β

Hypothetically disrupted coding sequences (HDCS) due to frameshift mutations or premature stop codons were determined in high quality finished and closed genome sequence of 11 representative strains from major subclades (Fig 1B and S3 Table). Representative strains of clade α generally contained fewer HDCS than those from clade β, with the exception of NCTC13348 (DT104) and SL1344 (DT204/49) that had atypically high and low numbers of HDCS, respectively (Fig 1B). However, none of the HDCS in NCTC13348 were in genes previously been implicated in pathogenesis, while SL1344 contained two virulence gene HDCS (*lpfD* and *ratB*) (Fig 1A). In general, clade α strains had 0–3 HDCS in virulence genes (mean 0.8 SD 0.9), while clade β was characterised by multiple lineage containing three or more virulence gene HDCS (mean 5.0 SD 3.2) (Fig 1A and S4 Table). Isolates in clade β5 (DT56, passerine bird associated [13]) contained up to eleven virulence gene HDCS (*lpfD*, *ratB*, *sseK3*, *siiE*, *siiC*, *ttrB*, *sseJ*, *gogB*, *sseK2*, *fimH* and *katE*). The greatest number of HDCS in clade α were observed in α12 (U288, possibly pig adapted), in which three virulence genes were affected (*avrA*, *sadA* and *tsr*). *LpfD* was found to be the only HDCS (S3 Table) that segregated between clade-α and clade-β. A 10-nucleotide deletion causing a frameshift mutation in *lpfD* resulting in a truncation approximately half way into the protein in all isolates of clade-β.

To quantify the relative level of functional divergence in the proteome of isolates in each clade we used a profile hidden Markov Model approach, delta-bitscore (DBS profiling) [33] (Fig 1C and S5 Table). The method assigned a value (bitscore) to peptides of the proteome that indicated how well each sequence fitted the HMM. We determined the difference in bitscore of the proteome of each isolate relative to that of *S*. Typhimurium strain SL1344 (DBS = bitscore SL1344 proteome—bitscore test proteome). A greater DBS is therefore indicatives of excess of polymorphisms that potentially alter protein function, and most likely a loss of function as it indicates divergence from the profile HMM. Mean DBS was significantly greater (p<0.05, Wilcoxon test) for proteomes of strains in clade-β compared with clade-α (Fig 1C). In general, third-level clades in clade-α exhibited DBS of approximately zero, consistent with limited functional divergence. Notably, despite considerable numbers of HDCS in strain NCTC13348 (α15, DT104), DBS was only moderately elevated in this clade. Proteomes of strains in clade β exhibited mean DBS of 0.03 and above with the exception of clades β1 and β2. The proportion of the proteome with any deviation in DBS was also greater in clade-β than clade-α (Fig 1D).

We also used a machine learning approach to predict the ability of strains to cause extraintestinal disease based on convergent patterns of mutation accumulation detected by delta bitscore (DBS), in 196 proteins that were recently determined and reported as the most predictive of the invasiveness phenotype in 13 serotypes (six extra-intestinal pathovars and seven gastrointestinal pathovars) of *S*. *enterica* subspecies I [34, 35] (Fig 1E). Protein sequences for the 196 genes were retrieved for each isolate in the study, scored by DBS, and run through the model. The invasiveness index metric is the fraction of decision trees in a random forest algorithm that vote for an invasive phenotype based on DBS values. The invasiveness index was significantly greater (p<0.05, Wilcoxon test) in clade-β than clade-α, consistent with the epidemiology and pathogenicity of the isolates located in these clades [3, 4].

### The clade-specific accessory genome is largely driven by acquisition of prophage genes and integrative elements

**Fig 2:**
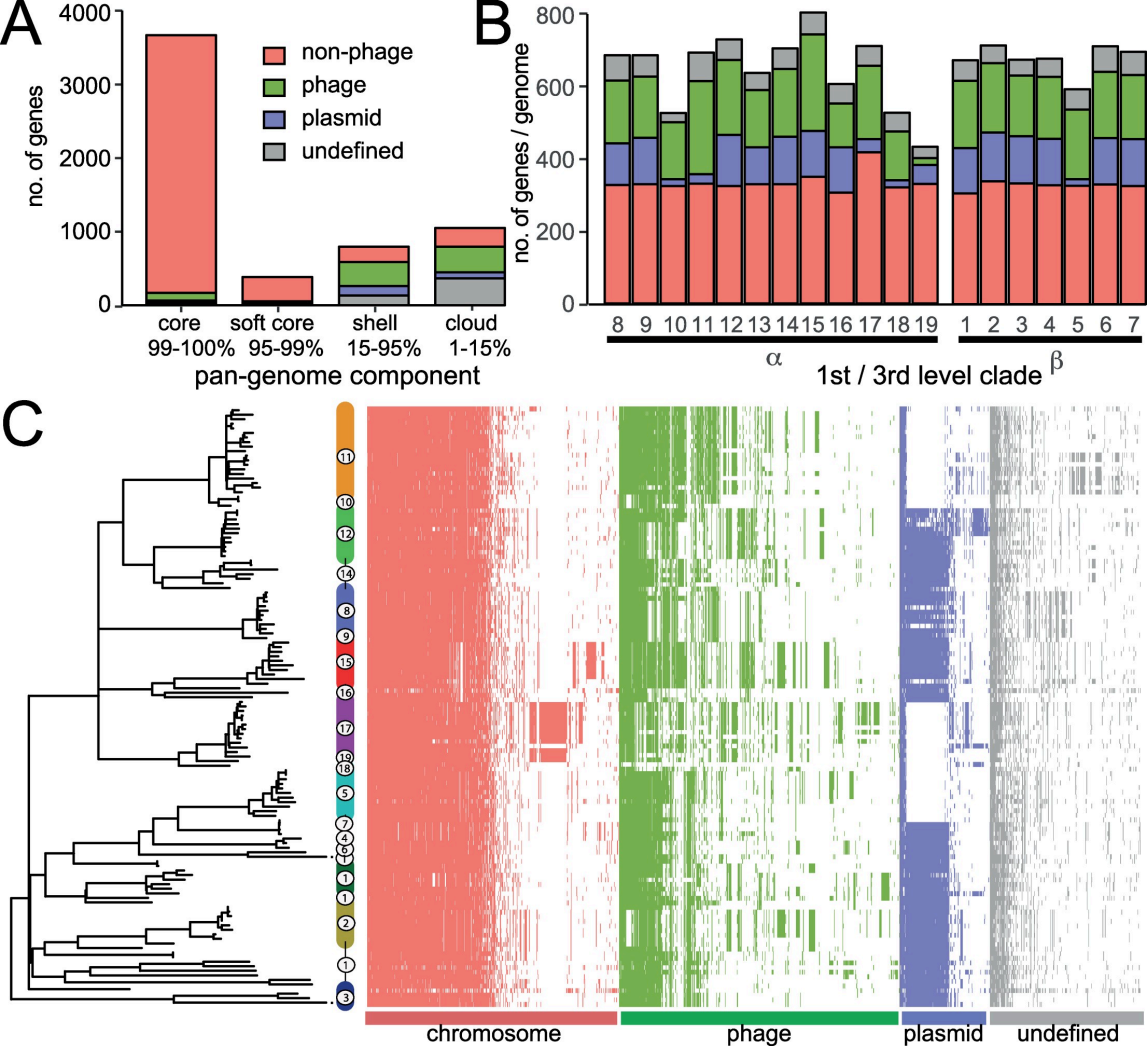
The pan genome of 131 *S*. Typhimurium isolates. Gene families were identified based on sequence alignment with a cut off of 90% sequence identity and assigned to non-prophage chromosomal (red), prophage (green), plasmid (blue), or undefined (grey), based on their genome context in eleven annotated reference genomes from each third level clade. (A) Number of genome families in the core, softcore, shell and cloud components of the pangenome. (B) Number of genome families of each pan genome component in isolates from each *S*. Typhimurium third level clade. (C) Accessory genome (shell and cloud) in each isolate. Gene families present in more than 130 or less than 5 strains were excluded. Maximum Likelihood tree based on variation (SNPs) in the core genome with reference to S. Typhimurium SL1344. Third-level clades are indicated in colour coded in common with the phylogeny vertical bars.

A pangenome analysis of *S*. Typhimurium (excluding the ST36 phylogroup) identified 9167 total gene families. The core genome (present in 99–100% of strains) was 3672 genes, soft core genome (95–99%) 388 genes. Shell genome (15–95%) 792 genes, and cloud genes (0–15%) 4315 genes (Fig 2A and 2B). We defined gene families of the accessory genome as non-prophage chromosomal, prophage, plasmid and undefined, based on their location and annotation in the complete and closed genomes of eleven reference strains phylogenetically distributed across S. Typhimurium (Table 1). Gene families not present in reference genomes were classified as ‘undefined’. The accessory genome was defined as genes present in 95% or fewer of isolates and thus represents the major source of genetic variation between strains.

Some gene families exhibited a distinct distribution in clade α or β, or within individual third-level clades of clade α or β (S4 Fig). Four non-phage chromosomal genes were specifically associated with clade-β strains, STM0038 a putative arylsulfatase, *tdcE* encoding a pyruvate formate lyase 4, *aceF* acetyltransferase and *dinI* a DNA damage inducible protein. A series of plasmid genes in clade β2 corresponded to a region of p2 seen in LO1157-10. This region is likely a transposon as it contains an IS200 transposase and an integrase. Also, a number of prophage-associated genes were present throughout clade-β due to apparent recombination in the ST64B prophage. The rate of gene flux in clade α and clade β was determined by computing the number of accessory genes as a function of SNPs in each clade. By this measure, the rate of gene flux was nearly twice as high in clade α compared to clade β (S5 Fig).

Generally, gene flux in the non-prophage and non-plasmid gene families that were specific to individual third level clades was limited to individual genes or small blocks of genes (S4 Fig). The exception was two large genetic islands in clades α15 (DT104 complex) and α17 (monophasic *S*. Typhimurium ST34) corresponding to the presence of SGI1 [36] and SGI4 [37]. In addition, a chromosomal block of genes in clade β2 corresponding to an insertion at the Thr-tRNA at 368274 containing a series of hypothetical proteins and a gene with similarity to the *trbL* gene involved in conjugal transfer (A0A3R0DZN6) and a site-specific integrase. Some of these genes were also present in isolates in clades β1 and β3. The greatest contribution to third level clade specific gene families was in the those with predicted functions in prophage (Fig 2C and S4 Fig).

### Extant prophage repertoire is the result of recombination and infrequent loss of ancestral elements and acquisition of new phage

**Fig 3:**
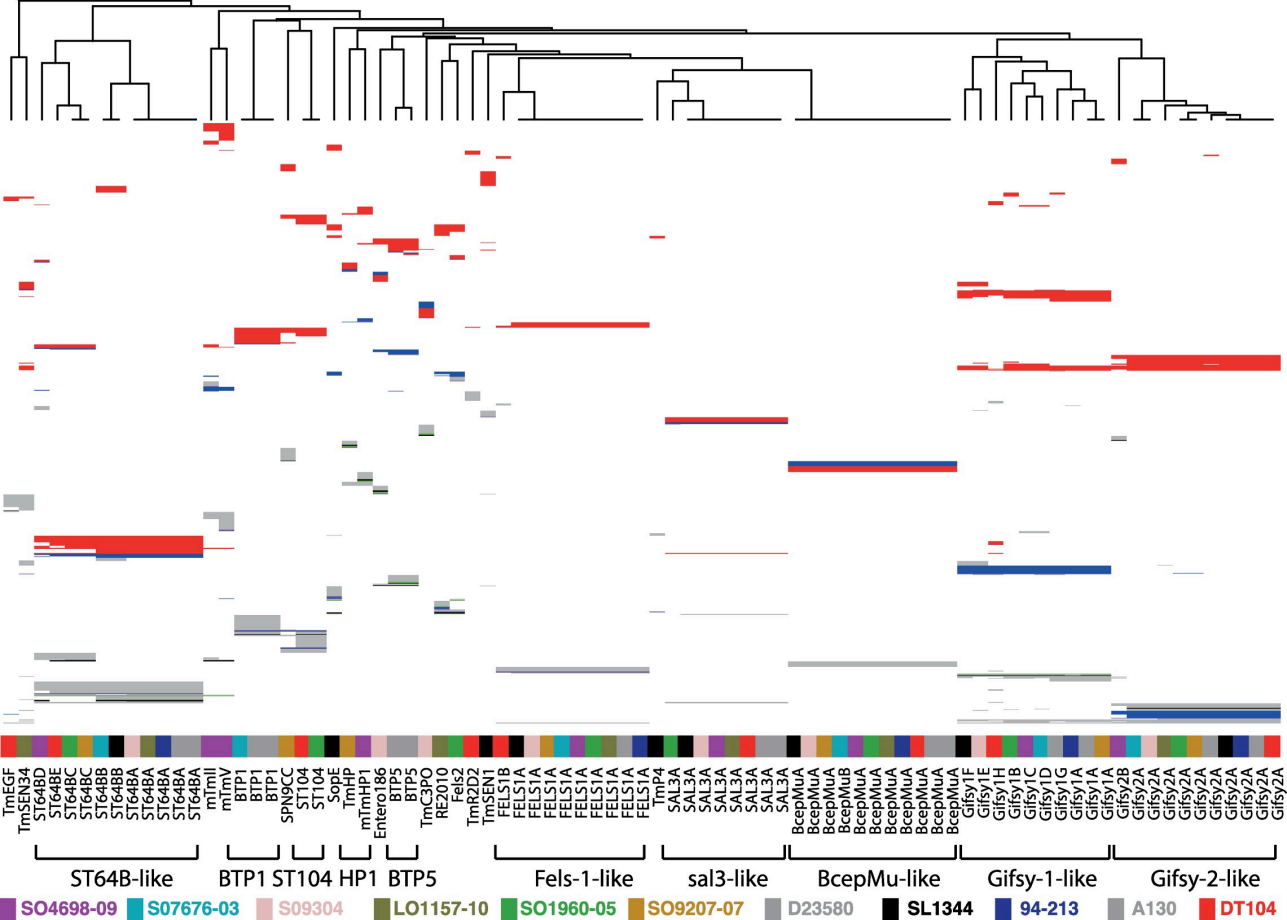
Clustering of prophage genes based on sequence identity indicates related families and potential recombination. Genes from all prophage identified in complete and closed whole genome sequence of eleven reference strains of *S*. Typhimurium were assigned to families based on sequence identity (>90% identity). Prophage genes (columns) were clustered to identify related prophage. The presence of a gene is indicated with a box predicted function based on *in silico* annotation are colour coded based on annotation, terminase (black), capsid (green), recombinase/integrase (purple), tail fibre (blue), other phage associated (red), and hypothetical protein (grey). A cladogram showing the relationship of prophage is based on the pattern of gene presence or absence is indicated (top).

**Fig 4:**
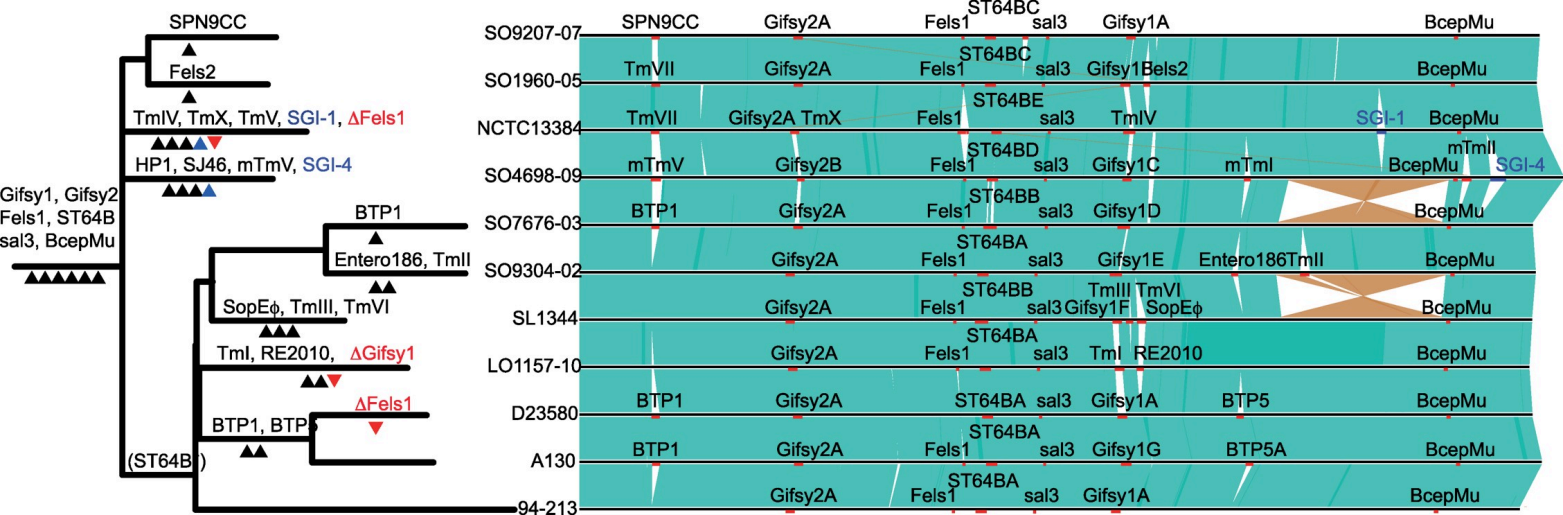
Genome alignment and phylogenetic relationship of complete and closed reference strains of *S*. Typhimurium or used in this study. Sequence with >90% nucleotide sequence identity are indicated where this is direct alignment (green) or reverse and complement (red). The location of prophage sequence (red bars) or integrative elements (blue bars) are indicated. A maximum likelihood tree based on sequence variation (SNPs) in the core genome with reference to *S*. Typhimurium strain SL1344 (left) is annotated with the most likely order of acquisition (black arrow) or loss (red arrow) of prophage and integrative elements, based on the principle of parsimony.

In order to investigate the flux of prophage genes resulting in clade-specific repertoires, we identified prophage in eleven complete and closed reference genomes of *S*. Typhimurium sequences. A total of 83 complete or partial prophage elements were identified in the eleven reference genomes (Fig 3). Prophage were present at twelve variably occupied chromosomal loci and the number per strain ranged from five in DT2 (strain 94–213) to nine in monophasic *S*. Typhimurium ST34 (strain SO4698-09) (Table 1). Clustering of gene families in the prophage pangenome identified 23 prophage, although in some cases blocks of genes were replaced resulted in mosaic prophage for example “*Salmonella* virus ST64BX” and “*Salmonella* virus Gifsy1X” (S6 Table), and the definition of families of prophage with a high confidence was consequently problematic (Fig 4). Thirteen prophage elements encoded at least one identifiable cargo gene, capable of modifying the characteristics of the host bacterial strain, including eleven genes previously implicated in virulence (S3 Table). Ten prophage families contained no recognisable cargo genes. The evolutionary history of prophage acquisition and loss was reconstructed based on principles of maximum parsimony. Six prophage (*Salmonella* viruses “BcepMuX”, “Gifsy1X”, “Gifsy2X”, “Fels1X”, “ST64BX” and “sal3X”, hereafter referred to as BcepMu, Gifsy1, Gifsy2, Fels1, ST64B and sal3) (S6 Table), that together accounted for 61 of the prophage in these genomes, were most likely present in the common ancestor of *S*. Typhimurium. Loss of two of these ancestral prophage by three isolates (NCTC13348, L01157-10 and D23580) represented the only evidence for decrease in prophage repertoire in the dataset.

A total of 22 additional prophage had a limited distribution within *S*. Typhimurium strains, present in three or fewer genomes *Salmonella* viruses (“TmEGF”, “TmSEN34”, “mTmII”, “mTmV”, BTP1, “TmST104”, “SPN9CC”, Fels2, “TmHP1/mTmHP1”, BTP5, “TmC3PO”, “RE2010”, “TmR2D2” and “TmSEN1”) (S6 Table) and are therefore likely to have been acquired during the evolution of *S*. Typhimurium (Fig 4). *Salmonella* virus BTP1 that was reported to be specific to the ST313 strains associated with epidemics of invasive NTS disease in sub-Saharan Africa [38], was also present in strain SO7676-03, a strain in clade β5 (DT56 complex) adapted to circulation in wild bird (Passerine) species [12]. “*Salmonella* virus mTmV” of strain SO4698-09, that carries the *sopE* virulence gene in some monophasic *S*. Typhimurium ST34 isolates [18], was absent from all other *S*. Typhimurium reference strains. However, a second prophage “*Salmonella* virus mTmII” with similarity to SJ46 was also in strain SO4698-09, and shared several clusters of gene families in common with mTmV.

**Fig 5:**
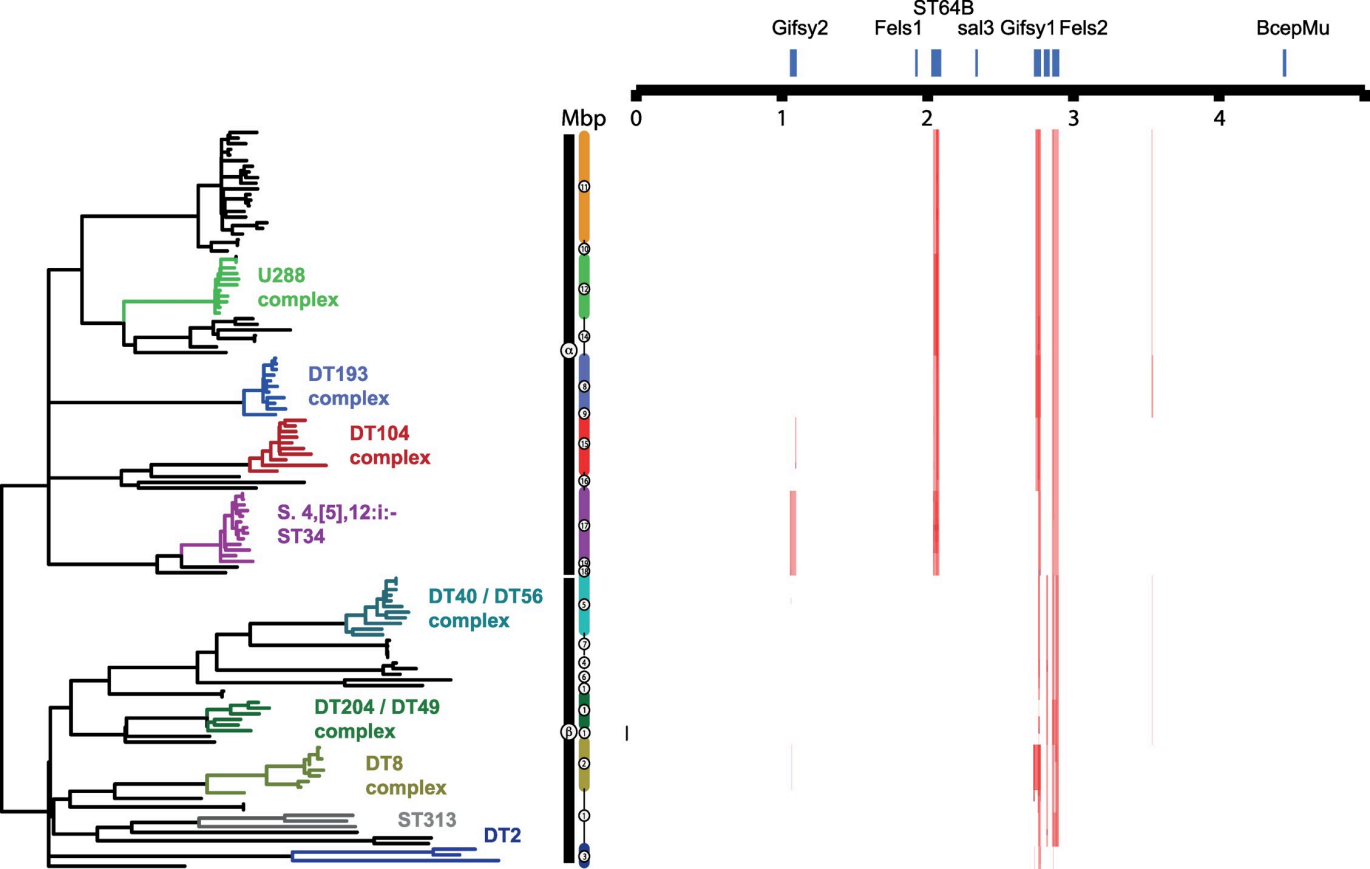
Recombination inferred by high SNP density in 131 *S*. Typhimurium strains. Regions of high SNP density (red) are indicated for each of the 131 isolates in the *S*. Typhimurium ST19 cluster with reference to the *S*. Typhimurium strain SL1344 genome. Recombination is shown with reference to the population structure and phylogeny of Typhimurium shown in Fig 1. The position of predicted prophage (blue) in the *S*. Typhimurium strain SL1344 genome are indicated (top).

With the notable exception of BcepMu the prophage predicted to be present in the common ancestor of *S*. Typhimurium exhibited considerable variation, potentially due to recombination [39] (Fig 4). Recombination is a major source of genetic variation in bacteria, although the level of recombination seen in other bacteria and indeed other *Salmonella* serovars vary greatly. We identified potential recombination in the genome sequence of the 131 *S*. Typhimurium of the main phylogroup by the identification of atypical SNP density. Recombination was almost exclusively present in prophage regions resulting in clade specific sequence variation (Fig 5). Recombination resulted in replacement of large blocks of gene families in ancestral prophage elements. Fels1, sal3 and Gifsy2 were conserved in the most reference strains, with the exception of Fels1 in DT104 and Gifsy2 in monophasic *S*. Typhimurium ST34 that had large alternative blocks of gene families. Gifsy1 was highly variable in all strains, but retained a core set of genes suggesting common ancestry and frequent recombination. Variation in ST64B was also present in most strains, and variable blocks of genes distinguished strains in first order clades α from β, and resulted in the acquisition of *sseK3* virulence gene by the common ancestor of the latter.

## Discussion

The population structure of *S*. Typhimurium consisted of two relatively distantly related clusters comprising strains of ST36 and a second (main) phylogroup predominantly ST19, containing the remainder of *S*. Typhimurium, consistent with previous reports of two distinct *S*. Typhimurium phylogroups [40, 41]. These two groups were more closely related to each other than to other serotypes of *S*. enterica subspecies I. The main Typhimurium phylogroup exhibited a star shaped phylogeny with multiple deeply rooted branches emerging from a common ancestor, with diversification at the terminal branches in some cases, associated with expansion of epidemic clonal groups. The topology of this *S*. Typhimurium phylogroup was similar to that of serotypes of *S*. *enterica* subspecies I [42, 43], with internal branches radiating from a common ancestor, defined by the accumulation of hundreds of SNPs in *S*. Typhimurium compared with tens of thousands of SNPs defining lineages of representative strains of distinct serotypes of *S*. enterica subspecies I [4].

The nested phylogenetic structure, rooted with the *S*. Heidelberg outgroup, was characterised by two high order clades (α and β), in which clade α was basal to clade β. Several deeply rooted lineages of clade α contained isolates almost entirely from livestock. A single lineage originating from the common ancestor of the main *S*. Typhimurium phylogroup gave rise to the common ancestor of clade β and diversification into multiple lineages was accompanied by apparent host adaptation to diverse host species, but notably many more avian species, compared with clade α. The β subclades included those associated with the DT56, DT2 and DT8 complexes that are well characterized host adapted clonal groups [11, 12, 21], contained exclusively isolates from avian species, and were present on relatively extended internal branches. This general phylogenetic topology is consistent with that described for distinct collections of *S*. Typhimurium strains from North America and Asia [44, 45], and Enterobase [24]. The EnteroBase database contained over 40,000 *S*. Typhimurium genome sequences at the time of writing, with additional additional α and β subclades not present in our dataset, presumably because they are not present in the UK. A lack of isolates from wild avian hosts and incomplete metadata limited our ability to test many of our key findings with this larger dataset.

Isolates in α subclades and some β subclades were under distinct anthropogenic selection pressure for the acquisition and maintenance of AMR, that correlated with their distribution in livestock or wild avian species [46]. Clade α isolates that were predominantly from livestock contained several lineages with multiple AMR genes, while most clade β isolates contained few or no AMR genes. Differential selection pressure for acquisition and maintenance of AMR genes is consistent with the idea that some *S*. Typhimurium genotypic variants are adapted to circulation in specific host populations that exert different selection pressure for the acquisition and maintenance of AMR genes. Antimicrobials have been used widely to control infection or as growth promoters in livestock, but wild animals are unlikely to encounter therapeutic levels of these drugs [47]. However, *S*. Typhimurium strains of DT56 and DT40 present in clade β and known to be associated with passerine birds, lacked AMR genes, yet are occasionally isolated from cattle and human clinical infections where they may be subject to selection for antimicrobial resistance [48]. We might therefore also expect to find AMR genes in DT56 and DT40 strains. One possibility is that DT56 and DT40 strains from clade β may transiently colonise the cattle host but are unable to circulate in this population and do not transmit back to the avian population with high frequency. Host adaptation to avian species therefore appears to create an effective barrier to circulation in livestock. Two clade β lineages did contain strains with multiple AMR genes, but in each case their epidemiology was atypical for clade β in that they were associated with an epidemic in cattle (DT204/49 complex) or invasive NTS in people in sub-Saharan Africa (ST313) [7, 22], and therefore were likely to be under selection for AMR.

The molecular basis of the barrier to circulation of some clade β isolates in livestock is not known, but likely the result of genotypic changes affecting functional diversification of the proteome. The proteome delta bitscore (DBS) of clade β isolates exhibited elevated divergence from profile HMMs of protein families in gamma proteobacteria, compared to clade α isolates, potentially resulting in loss or altered protein function [21]. Similarly, divergence was reported in the *S*. Gallinarum proteome, a serotype highly host adapted to poultry where it is associated with fowl typhoid [33]. β subclades also exhibited an elevated invasiveness index, the fraction of decision trees using random forests that vote for the invasive (extraintestinal) disease outcome, a predictive score of host adaptation to an extraintestinal lifestyle. Consistent with this finding, increased invasiveness of strains from clade-β lineages has been reported previously, including *S*. Typhimurium DT2 isolates in day of hatch chicks [21] and ST313 isolates in day of hatch chicks [49]. Signatures of invasiveness of ST313 strains was also consistent with changes in interaction with mice and cattle in experimental models of infection [50, 51], and multicellular behaviour in the environment [52].

We used a machine learning approach to determine an invasiveness index that was not designed to identify mechanism of invasiveness, but instead discriminated genotypes associated with alternative pathotypes on the basis of shared proteomic signatures [34]. The relationship of invasiveness index and site of isolation was reported previously [53]. In our analysis DBS of 196 protein families most predictive of disseminated disease in *S*. Typhi, *S*. Patatyphi A, *S*. Gallinarum, *S*. Dublin and *S*. Choleraesuis, also predicted an extraintestinal lifestyle in *S*. Typhimurium β subclades, consistent with the epidemiological data [4]. The pattern of functional divergence in some *S*. Typhimurium β subclades may therefore at least in part be by a process of convergent evolution with that observed as a result of the evolution of several extraintestinal serotypes *S*. *enterica*, including *S*. Typhi, *S*. Patatyphi A, *S*. Gallinarum, *S*. Dublin and *S*. Choleraesuis [34].

Host adaptation of bacteria is commonly associated with the accumulation of HDCS, potential pseudogenes that contribute to genome degradation [54, 55]. A total of 24 genes previously implicated in virulence, adhesion or multicellular behaviour were HDCS in one or more clade β isolates. Notably, 15 of these genes (63%) were also HDCS in highly host adapted *S*. Typhi or *S*. Paratyphi A, serotypes that are restricted to humans and cause a disseminated disease[56]. HDCS were especially common in strains of DT56/DT40 complex (clade β5), that are reported to be highly host adapted to passerine birds, in which eleven HDCS were observed. In *S*. Paratyphi A, many mutations and gene flux that occurred was reported to be neutral, indicated by their sporadic distribution within clades, and frequent loss from the population by purifying selection [57]. In *S*. Typhimurium, we also observed some examples of potential neutral mutations, but in many cases HDCS in virulence genes were present in multiple related strains from the same subclade, indicating that they were likely under selection, and stably maintained in the population (Fig 1). In contrast to clade β, genome degradation affecting virulence-associated genes was less frequent in isolates clade α. Just three virulence gene HDCS had a clade phylogenetic signature, *avrA*, *tsr* and *sadA*, in α10, α11 and α12. Subclade α12 (U288 complex) was the only clade to contain all of these HDCS, consistent with the U288 complex exhibiting apparent host-adaption to pigs [17, 37]. Therefore, the presence of HDCS in virulence associated genes was almost exclusively associated with subclades containing strains with strong epidemiological evidence of host adaptation [4]. Despite the *S*. Typhimurium DT104 complex strain NCTC13348 (clade α15) exhibiting a high level of genome degradation that was uncharacteristic for α subclades and the broad host range epidemiology of the clonal group [58], no virulence or multicellular behaviour genes were HDCS [58]. Furthermore, the mean DBS for the proteome of strains from clade α15 was similar to that of other α subclades, suggesting that functional divergence as a whole was not atypical from that of other clade α isolates.

While genes encoding components of the type III secretion systems (T3SS) 1 and 2 apparatus were never HDCS, several genes encoding effector proteins secreted by them were (*sseI*, *sseK2*, *sseK3*, *avrA*, *sseL*, *sseJ* and *gtgE*). The *sseI* gene is inactivated in ST313 due to insertion of a transposable element, and results in hyper dissemination of these strains to systemic sites of the host via CD11b^+^ migratory dendritic cells [51]. The *sseK2*, *sseK3*, *avrA* and *sseL* genes each encode effectors that inhibit the NFκB signalling pathway thereby modulating the proinflammatory response during infection [59–61]. Furthermore, these effectors are commonly absent or degraded in serotypes of *Salmonella* serotypes associated with disseminated disease [62, 63], suggesting that altered interaction with the macrophage is essential for disseminated disease in diverse *Salmonella* variants and hosts. In addition, several genes encoding components of fimbrial or afimbrial adhesin systems (*sadA*, *ratB*, *lpfD*, *stfD*, *stbD*, *safC*, *fimH*, *siiC* and *siiE*), or anaerobic respiration (*ttrB*), and chemotaxis (*tsr*) were HDCS in one or more isolates. Many of these genes have been implicated in intestinal colonisation [31, 64–69], suggesting that their inactivation in host adapted variants may be associated with a loss of selection for functions no longer required in a reduced host range or where intestinal colonisation is no longer critical to transmission. The *sadA* and *katE*, genes are involved in multicellular behaviour, biofilm formation and catalase activity that protects against oxidative stress during high density growth functions, respectively, and are commonly affected by genome degradation in host adapted pathovars of *Salmonella* [52, 70].

The only virulence-related HDCS that segregated clades α and β resulted from a 10 bp insertion in the *lpfD* gene of all clade β isolates. Within the host *S*. Typhimurium preferentially colonises Peyer’s patches (PPs) [71], due to long-polar fimbriae *Lpf* binding to M-cells [72]. Similarly, *lpf* genes in *E*. *coli* pathotypes are required for interaction with Peyer’s patches and intestinal colonisation [73]. Despite the disruption of *lpfD* in the clade β isolate SL1344, deletion of *lpf* reduced colonisation on the surface of chicken intestinal tissue, [74], suggesting that long polar fimbriae retain function. Differences in intestinal architecture of avian species that have the lymphoid organ the bursa of Fabricius containing numerous M cells compared with mammalian species that have Peyer’s patches with relatively scarce M cells [75, 76] may explain the pattern of *lpfD* HDCS in S. Typhimurium. The function of long polar fimbriae expressing full length LpfD in clade α isolates has not been investigated, but its distribution in isolates from livestock and human infections mark it as of potential importance to human health. Other virulence genes including *gtgE*, *avrA* and *sseK3*, exhibited sporadic distribution or HDCS within third level clades, consistent with observations in models of infection suggesting that they may play a role in adaptation to an invasive lifestyle [77–80].

Bacterial genome diversity is largely driven by the flux of genes resulting from acquisition by horizontal gene transfer and deletion, rather than allelic variation [81]. The accessory genome of *S*. Typhimurium revealed few genes that segregated clade α and β, but distinct forms of ST64B prophage resulting from recombination that replaced a large block of genes were present in clade α and β, and resulted in the presence of *sseK3* specifically in clade β. The accessory genome contributed significantly to genetic variation that distinguished third order subclades in both clade α and β, especially phage and plasmid genes. Non-phage chromosomal genes exhibited relatively little clade specific accessory genome suggesting that the majority was the result of deletions or gene acquisition on small mobile genetic elements that were neutral and subsequently lost, as observed previously in *S*. Paratyphi [57]. However, three large genetic elements were acquired on the chromosome in α15 (DT104) or α17 (monophasic *S*. Typhimurium ST34), the two most recent dominant MDR pandemic clonal groups that together account for over half of all S. Typhimurium infections in the human population Europe in the past 30 years. The acquired genes corresponded to SGI1 [36] in the DT104 complex, and SGI4 and a composite transposon in monophasic *S*. Typhimurium ST34 [18, 37], highlighting the likely importance of horizontal gene transfer in the emergence of epidemic clones.

Variable prophage repertoires are a major source of genetic diversity in *Salmonella* [82, 83], and may contribute to the emergence and spread by impacting the fitness during intra-niche competition due to lytic killing or lysogenic conversion of competing strains [84]. This view was supported by the considerable phage-associated gene flux observed in *S*. Typhimurium. Importantly, the phage component of the accessory genome in *S*. Typhimurium had a strong correlation with the third level clade, suggesting that although transfer was frequent, the acquisition or loss of prophage elements was not transient, consistent with selection within each clonal group [57]. Reconstruction of the evolutionary history of prophage elements in the main *S*. Typhimurium phylogroup indicated that the common ancestor likely contained six prophage, Gifsy1, Gifsy2, Fels1, ST64B, sal3 and BcepMu, that were well conserved during subsequent diversification. Just two of these ancestral prophage, Fels1 and Gifsy1, were lost from the genome, on three occasions in different lineages. The majority of the prophage flux was from the acquisition of between one to three prophage in each lineage, with the exception of a lineage containing clade β3 (DT2), that only contained the ancestral prophage repertoire.

Together, our analyses are consistent with the view that the common ancestor of the main *S*. Typhimurium phylogroup was a broad host range pathogen with little genome degradation capable of circulating within multiple species of livestock. The age of the common ancestor of *S*. Typhimurium is not known and previous attempts to calculate this using Bayesian approaches have been frustrated by a weak molecular clock signal [85]. However, the common ancestor of *S*. Paratyphi A was estimated to have existed approximately 500 years ago and provides a frame of reference [57]. The main *S*. Typhimurium phylogroup exhibited greater genetic diversity than reported for *S*. Paratyphi A, with an estimated maximum root to tip SNP accumulation of approximately 750 and 250, respectively. Therefore, the common ancestor of the main *S*. Typhimurium phylogroup is likely to have existed earlier than that of *S*. Paratyphi A. However, the S. Typhimurium most recent common ancestor (MRCA) is unlikely to have predated the domestication of livestock, that began around ten thousand years ago [86], raising the possibility that the emergence of this phylogroup was linked to the anthropogenic selection provided by entry into a niche within the domesticated livestock. Subsequent to the emergence of the common ancestor of this phylogroup, a single lineage appears to have spawned multiple lineages, some of which have become highly host adapted to various wild avian species, by a process of convergent evolution with that observed in host adapted serotypes of *Salmonella* such as *S*. Typhi.

## Materials and methods

### Bacterial strains and culture

*S*. Typhimurium isolates and Illumina short read sequence used in this study have been described previously [87], selected based on phage type determined during routine surveillance by Public Health England (PHE) and the Animal and Plant Health Agency (APHA) in order to represent the diversity *S*. Typhimurium phage types as a proxy for genetic diversity. A strain collection of 134 *S*. Typhimurium or monophasic variant isolates was composed of 2 to 6 randomly selected strains from the top ten most frequent phage types from PHE and the top 20 most frequent phage types from APHA surveillance, from 1990–2010 (2–5 strains of each) were used in this analysis. In addition, commonly used lab strain SL1344 [88], two reference strains of ST313 (D23580 and A130)[89] and three DT2 strains isolated from pigeon [90] were included. For routine culture, bacteria were stored in 25% glycerol at -80^o^c and recovered by culture on Luria Bertani agar plates, and single colonies were selected to inoculate LB broth that was incubated at 37 ^o^c for 18 hours with shaking.

### Short-read *de novo* assembly

Illumina generated fastq files were assembled using an in-house pipeline adapted from that previously described [91]. For each paired end reads, Velvet [92] (1.2.08) was used to generate multiple assemblies varying the k-mer size between 31 and 61 using Velvet Optimiser [93] and selecting the assembly with the longest N50. Assemblies were then improved using Improve_Assembly software [94] that uses SSPACE (version 3.0) [95] and GapFiller (version 1.0) [96] to scaffold and gap-fill. Ragout was used to order contigs [97] based on comparison to the long-read sequences. The finished genomes were then annotated using Prokka (version 1.11) [98].

### Long-Read sequencing using Pacbio and sequence assembly

DNA for long-read sequencing on the Pacbio platform was extracted from 10 ml of cultured bacteria as previously described [87]. Data were assembled using version 2.3 of the Pacbio SMRT analysis pipeline (https://smrt-analysis.readthedocs.io/en/latest/SMRT-Pipe-Reference-Guide-v2.2.0/). The structure of the initial assembly was checked against a parallel assembly using Miniasm [99] which showed general agreement. The Pacbio best practice for circularizing contigs was followed using Minimus [100] and the chromosomal contiguous sequence in each assembly was re-orientated to begin at the *thrA* gene. Illumina short read sequence data were used to correct for SNPs and indels using iCORN2 (http://icorn.sourceforge.net/). The finished sequences were then annotated using Prokka [98].

### Phylogenetic reconstruction and population structure analysis

The paired-end sequence files for each strain were mapped to the SL1344 reference genome (FQ312003) [88] using the Rapid haploid variant calling and core SNP phylogeny pipeline SNIPPY (version 3.0) (https://github.com/tseemann/snippy). The size of the core genome was determined using snp-sites (version 2.3.3) [101], outputting monomorphic as well as variant sites and only sites containing A,C,T or G. Variant sites were identified and a core genome variation multifasta alignment generated. The core genome of 134 *S*. Typhimurium (3686476 nucleotides) (S2 Table) contained 17823 variant sites. The core genome (3739972 nucleotides) of 131 *S*. Typhimurium non-ST36 contained 8382 variant sites. The sequence alignment of variant sites was used to generate a maximum likelihood phylogenetic tree with RAxML using the GTRCAT model implemented with an extended majority-rule consensus tree criterion [102]. The genome sequence of *S*. Heidelberg (NC_011083.1) was used as an outgroup in the analysis to identify the root and common ancestor of all *S*. Typhimurium strains as a previous study has indicated both Heidelberg and Saintpaul serovars to be appropriate [103]. HierBaps (hierarchical Bayesian analysis of Population Structure) [16] was used to estimate population structure using three nested levels of molecular variation and 10 independent runs of the optimization algorithm as reported previously [104]. The input for this analysis was the same SNP variant matrix for the 131 strains with reference to SL1344 that was used to generate the GTRCAT phylogeny above. Estimation of coverage for our dataset was by determination of CCs with fewer than 100 allelic variants in 1679 whole genome sequences from human clinical infections in the UK in 2014 to 2015, and approximately 20,000 non-UK *S*. Typhimurium whole genome sequences for which SRA accession numbers were available (accessed April 2020)[24]. The union of CCs and the proportion of sequences was reported.

### *In-silico* genotyping

The presence of antibiotic resistance, virulence and plasmid replicon genes in short-read data was determined by the mapping and local assembly of short reads to data-bases of candidate genes using ARIBA [105]. The presence of candidate genes from the resfinder [106], VFDB [107] and PlasmidFinder [108] databases was determined. Reads were mapped to candidate genes using nucmer with a 90% minimum alignment identity. This tool was also used to determine the presence of specific genes or gene allelic variants. The results of the ARIBA determination of the presence or absence of the *lpfD* gene were confirmed using SRST2 [109] setting each alternative form of the gene as a potential allele. SRST2 was also used to verify the ARIBA findings of the VFDB data set, as the presence of orthologous genes in the genome was found to confound the interpretation of results. Candidate SNPs in key genes associated with AMR were determined from the SNP matrix created by SNIPPY and used for the phylogeny reconstruction. Sites were then verified by interrogation of the assembled and annotated short-read sequences.

### Determination of hypothetically disrupted coding sequences (HDCS)

HDCS were identified in high-quality finished Pacbio sequences and previously published reference sequences by identifying putative altered open reading frames using the RATT annotation transfer tool [110]. The *S*. Typhimurium strains SL1344 annotation (accession no. FQ312003) was transferred to each assembled sequence and coding sequences identified as having altered length were manually curated by comparison of aligned sequences visualised using Artemis comparison tool (ACT) [111]. Genes that contained either a premature stop codon or a frameshift mutation were classified as HDCS. The identified HDCS were used to construct a database that could be used as a reference for SRST2 (above) to detect presence or absence in short-read sequence data. Alleles were called based on matching to 99% sequence identity and allowing one miss-match per 1000 nucleotides.

### Determination of Delta Bitscore

Illumina short-read sequences were mapped to the SL1344 reference genome and annotated using PROKKA and then analysed in a pairwise fashion against SL1344 using delta-bit-score (DBS), a profile hidden Markov model based approach [33] with Pfam hidden Markov Models (HMMs) [112]. The mean DBS per genome and percentage of genes with mutations in Pfam domains (non-zero DBS) are reported.

### Determination of Invasiveness Index

The invasiveness index [34] for each strain was calculated to scan for patterns of mutation accumulation common to *Salmonella* lineages adapted to an invasive lifestyle. To calculate the invasiveness index, Illumina reads were mapped to a core-genome reference using the snippy pipeline above, and annotated using PROKKA. Protein sequences were then screened using phmmer from the HMMER3.0 package [113] to identify the closest homologs to the 196 predictive genes used by the invasiveness index model. Missing gene sequences were set to NA, to account for the possibilities of being missed during sequencing or misannotated. These genes were then scored against profile hidden Markov models (HMMs) for these protein families from the Eggnog database [34] using hmmsearch [113], to test for uncharacteristic patterns of sequence variation. Bitscores produced in the comparison of each protein sequence to its respective protein family HMM were then used as input to the model.

### Phage location and cargo in assembled long-read sequence

The location of prophage elements in assembled long-read sequences and published reference genome was determined using PHASTER [114], which identified regions as being intact, questionable or incomplete. This yielded a total of 83 potential complete and partial sequences across the 11 representative strains. Prophage sequences were annotated using PROKKA that identified terminase, tail fibre, recombinase/integrase proteins capsid proteins, phage related proteins, and hypothetical proteins.

### Determination of recombination

Recombination was inferred by identifying regions of high SNP density from whole genome alignments of short-read data to SL1344, using Gubbins [115]. The results were visualised using Phandango [116] and related to the predicted prophage locations in the SL1344 genome. Similar results were obtained using maximum likelihood inference using clonal frame [117].

### Determination of the *S*. Typhimurium pangenome

The annotated assemblies of 131 predominantly *S*. Typhimurium ST19 isolates were used as the input to the pangenome pipeline ROARY [118]. The presence or absence of genes was determined without splitting orthologues. In order to characterise the contribution of prophage and plasmids to the pangenome, genes were assigned to one of four categories, non-prophage genes located on the chromosome, prophage genes, plasmid genes and undefined genes, based on the similarity to annotated genes of complete and closed whole genome sequence of eleven reference strains. Orthologous genes were identified based on > 90% nucleotide sequence identity using nucmer [119]. A core-genome reference sequence (genes present in at least 99% of reference strains), was also constructed and used to determine the invasiveness index.

### Estimation of gene flux rates

Genes were assigned a score based on their presence in strains within a specific clade. The clade gene score was compared to the score determined for strains outside of the clade to determine whether the gene was more prevalent within the clade than without. Genes were classed as associated with the clade if their score was greater than the mean plus two standard deviations of the non-cladal score (corresponding to the top 95% in a normal distribution).

The number of clade associate genes was compared with the number of SNPs associated with a clade (this gives a measure of evolutionary time) to determine the level of gene flux for the clade. The level of gene flux in the two first-level clades was then compared.

### Prophage classification

PHASTER[114] curated prophage sequences were classified into species and genus-level groupings based on the current criteria used by the Bacterial and Archaeal Viruses Subcommittee of the International Committee on Taxonomy of Viruses (ICTV)[120]. At the species level, genomes were clustered at 95% nucleotide identity over the whole genome length, meaning that two genomes belong to two different species if they differ in more than 5% of their genome. Clustering was performed with CD-HIT-EST at 95% nucleotide identity over 95% of the alignment length (99% of alignment length of shorter sequence)[121] and with Gegenees, a pairwise nucleotide comparison tool, using the accurate settings of 200 bp fragment size and 100 bp step size[122]. The Gegenees output was used in combination with vConTACT2[123] to classify the prophage sequences into new or existing genera. Briefly, coding sequences were predicted with PROKKA[98] and transformed into a table linking genomes and their encoding proteins. This table was used as input into vConTACT along with the Viral RefSeq database v85[124]. vConTACT then used Diamond[125], Markov clustering MCL[126] and ClusterONE[127] to predict viral clusters based on shared protein content. The output was visualised using Cytoscape[128]. Genera were defined as vConTACT viral clusters which shared a significant (>50%) nucleotide identity. The clusters were then compared to the current and pending ICTV taxonomic classification (ictvonline.org) using blast and vConTACT viral cluster output containing reference genomes and all prophage sequences were assigned to new or existing taxa.

### Assembly and comparison of pSLT plasmid sequence

Plasmid sequences were determined by assembling Illumina short-read sequences with spades-3.8.0 using the plasmidSPAdes algorithm [129] with varying with k-mer sizes of 31, 41 and 51. Contigs larger than 70 kb were then compared against the NCBI blast database to identify forms of pSLT.

### Determination of the distribution of representatives among the rest of the world

A collection of 14,478 genomes from all available *S*. Typhimurium in EnteroBase to represent the global diversity outside of the United Kingdom. These genomes met several criteria; they were predicted to be serovar Typhimurium using SISTR software [130], were not ST36, the country of isolation was listed as a country other than the "United Kingdom", and the genome was within the HierCC:1100 (cEBG) ‘2’ cluster group (S7 Table). These genomes were grouped according to their EnteroBase HierCC:100 cluster definition, which groups genomes together if the nearest neighbour is no more 100 cgMLST alleles different [24]. We then included representative genomes from this study and determined the proportion of defined cluster groups contained at least one of the representative genomes described here.

## Supporting information

S1 Fig Phylogenetic relationship of *S*. Typhimurium and diverse *S*. enterica serotypes. Mid-point rooted maximum likelihood phylogenetic tree based on the variation (SNPs) in the core genome of 18 strains of *Salmonella* Typhimurium and 14 representative strain of diverse *S*. *enterica* subspecies *enterica* serotypes, with reference to *S*. Typhimurium strain SL1344 genome sequence. *S*. Typhimurium strains (red lineages and text) are present in two clusters, composed of 15 strains with isolates that are ST19, ST34, ST313, ST98 and ST568 and three more divergent isolates of ST36. The phylogeny is rooted with respect to *S*. Heidelberg and was calculated using SL1344 as a reference to create a core-genome variant-site alignment and the GTRCAT model in RAxML.
(PDF)

S2 Fig Distribution of 131 non ST36 isolates in this study in UK clinical isolates and non-UK isolates from enterobase. Grapetree visualization based on EnteroBase cgMLST allele profiles, including of (A) 1,693 *S*. Typhimurium isolates from clinical infections in the UK in 2014 and 2015, and (B) 14,760 genomes selected as the global diversity of *S*. Typhimurium outside of the United Kingdom in Enterobase database. Nodes are colour coded by EnteroBase HierCC HC100 cluster groups. HierCC groups containing non ST36 isolates in this study yellow circles. Scale indicates number of cgMLST alleles.
(PDF)

S3 Fig Sequence variation in selected pathogenicity islands. The percent sequence variation including SNPs and deletions in SPI-1, SPI-2, SPI-3, SPI-4, SPI-5 and SPI-6 are indicated from 0% (green) to 0.5% (red).
(PDF)

S4 Fig Accessory genome with strong clade association. Gene families with a strong clade association in clade α or β (A), or in one of the third level clades (B). Maximum likelihood phylogenetic tree and based on sequence variation (SNPs) in the core genome with reference to *S*. Typhimurium strain SL1344 (left). Third-level clades are indicated and colour coordinated with that in Fig 1. Genes in each clade were assigned a score based on the number of strains containing the gene within the clade. This score was also calculated for the strains outside the clade. Clade associated genes were defined as genes that had scores greater than the mean plus two SD of the score for all other clades. Genes are colour coded based assignment to non-prophage chromosomal (red), prophage (green), plasmid (blue), or undefined (grey).
(PDF)

S5 Fig Gene flux rate metrics determined for non-singleton gene families first-level clades. (PDF)

S1 Table *S*. Typhimurium strain collection used in this study related to determine population structures. Table can be viewed at https://www.dropbox.com/sh/dh84yyc4tguirw3/AABnbbrSPtqEcjGY6IR6GggLa?dl=0.
(XLSX)

S2 Table Presence of plasmid operons, AMR genes (Resfinder) and virulence genes (VFDB) determined by *in-silico* genotyping using ARIBA software. The identifier column corresponds to the study-identifier column in S1 Table. Presence of genes are indicated by ‘1’. Table can be viewed at https://www.dropbox.com/sh/dh84yyc4tguirw3/AABnbbrSPtqEcjGY6IR6GggLa?dl=0.
(XLSX)

S3 Table Genome degradation in long-read reference strains. Potential hypothetically disrupted coding sequences (HDCS) were identified in reference genomes though anomalies in annotation transfer from the SL1344 reference sequence using RATT software and manual curation to exclude false positive HDCS. Table can be viewed at https://www.dropbox.com/sh/dh84yyc4tguirw3/AABnbbrSPtqEcjGY6IR6GggLa?dl=0.
(XLSX)

S4 Table Summary of HDCS in *S*. Typhimurium main phylogroup. The presence of HDCS alleles was determined *in-silico* using SRST2 reported in Fig 1. The study identifier refers to isolates in S1 Table. Alleles are specified as wild-type (WT) or HDCS. In some cases, multiple HDCS forms were determined to be present and these are denoted as HDCS1 or HDCS2 etc. Table can be viewed at https://www.dropbox.com/sh/dh84yyc4tguirw3/AABnbbrSPtqEcjGY6IR6GggLa?dl=0.
(XLSX)

S5 Table Summary of DBS and Invasiveness Index analysis. Isolate identifier corresponds to the identifier column in S1 Table. The mean delta bitscore (DBS) for the proteome of each strain, the number of proteins with a DBS greater than ten. Invasiveness index for each isolate is indicated.
(XLSX)

S6 Table Characteristics of prophage elements present in complete and closed whole genome sequence of *S*. Typhimurium reference strains. (DOCX)

S7 Table Data used for construction of HierCC clusters with 14,478 genomes from *S*. Typhimurium in EnteroBase to represent the global diversity outside of the United Kingdom. (XLSX)
