# Supplementary figures and images for: Evolution of Salmonella enterica serotype Typhimurium driven by anthropogenic selection and niche adaptation
Source: PLoS Genet. 2020 Jun 8;16(6):e1008850. doi: 10.1371/journal.pgen.1008850 (PMC7302871; doi:10.1371/journal.pgen.1008850)

S1 Fig

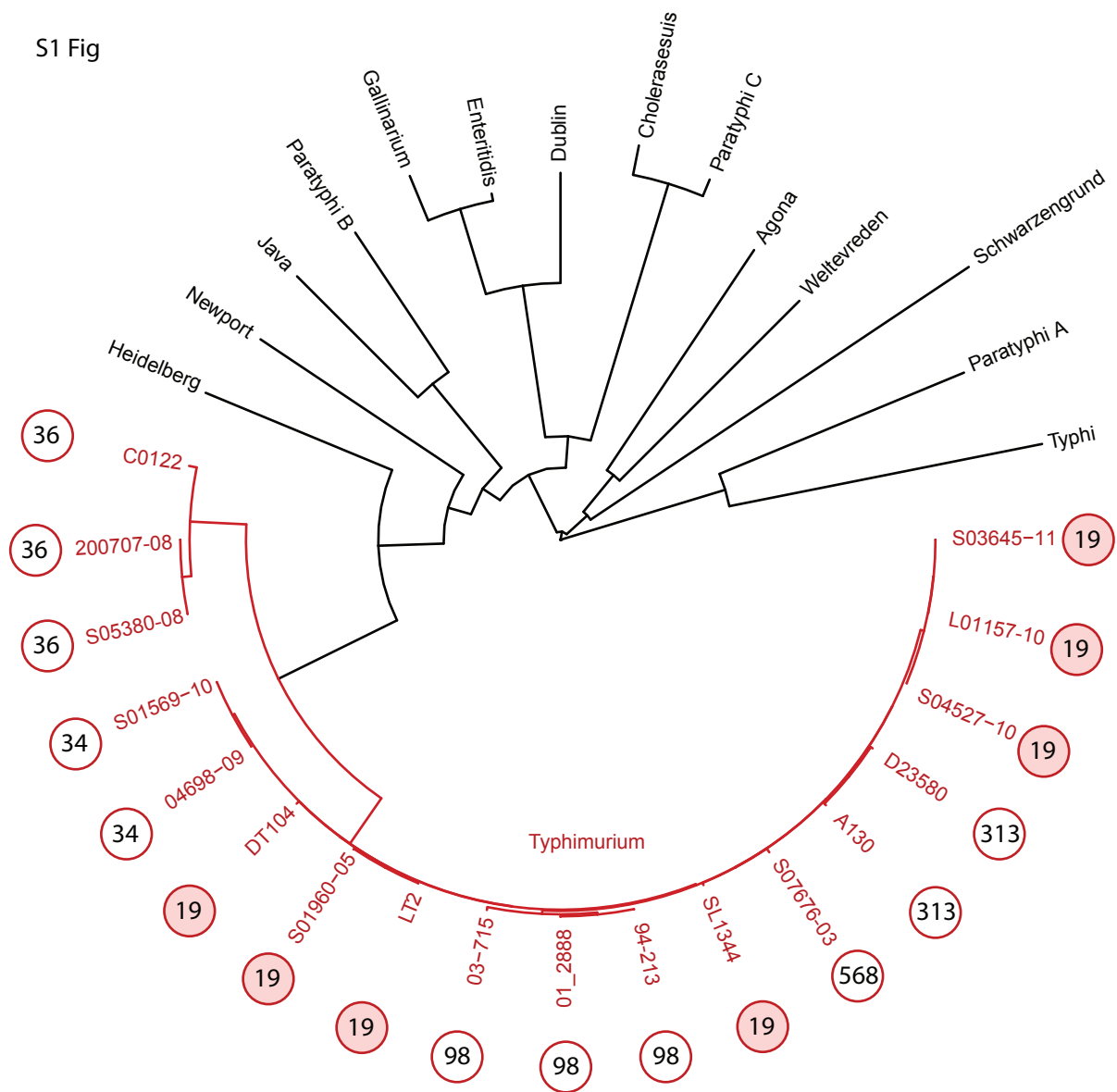

Supplement: S1 Fig — Mid-point rooted maximum likelihood phylogenetic tree based on the variation (SNPs) in the core genome of 18 strains of Salmonella Typhimurium and 14 representative strain of diverse S. enterica subspecies enterica serotypes, with reference to S. Typhimurium strain SL1344 genome sequence. S. Typhimurium strains (red lineages and text) are present in two clusters, composed of 15 strains with isolates that are ST19, ST34, ST313, ST98 and ST568 and three more divergent isolates of ST36. The phylogeny is rooted with respect to S. Heidelberg and was calculated using SL1344 as a reference to create a core-genome variant-site alignment and the GTRCAT model in RAxML. (PDF) [file pgen.1008850.s001.pdf]

A: PHE clinical isolates

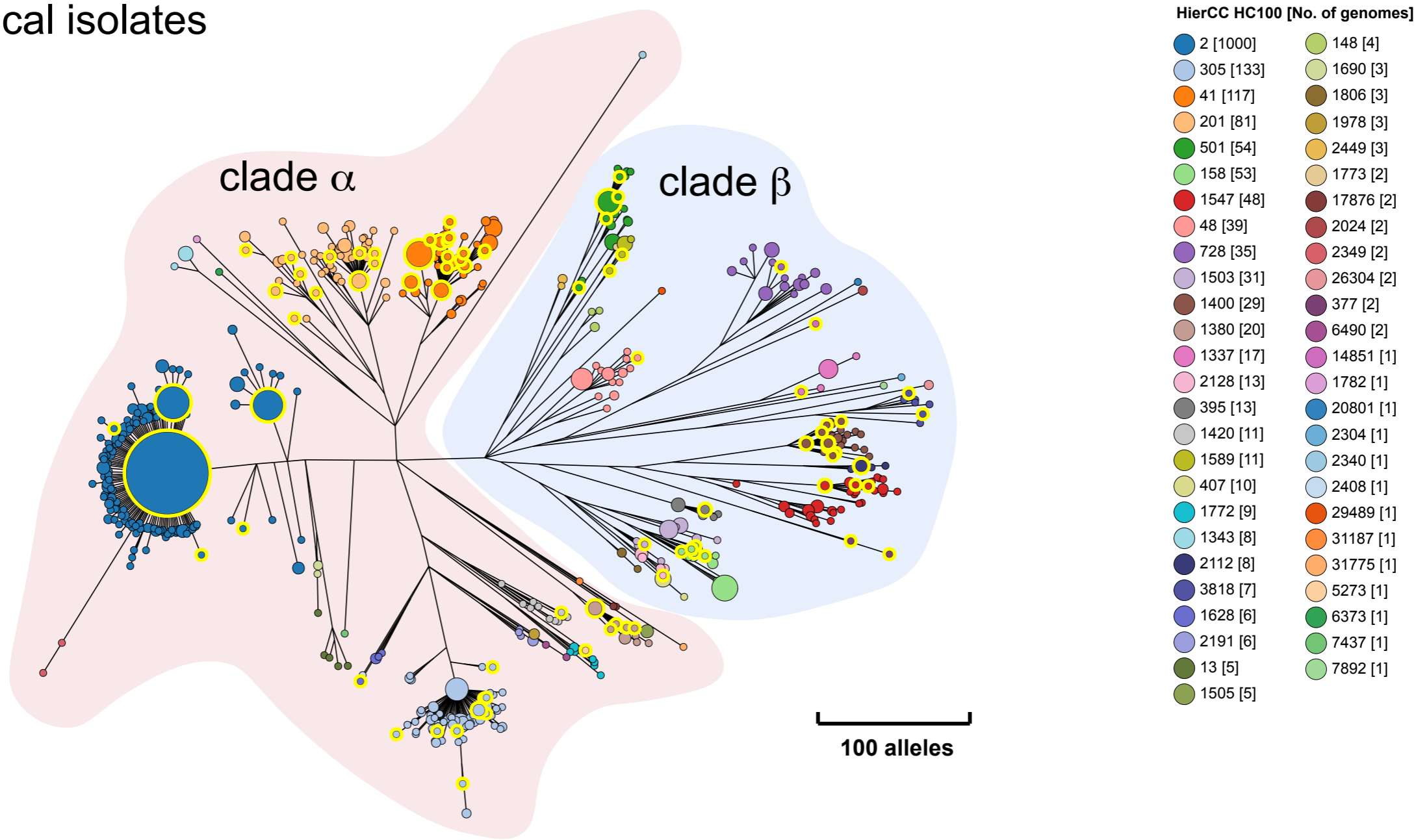

B: Non-UK Enterobase

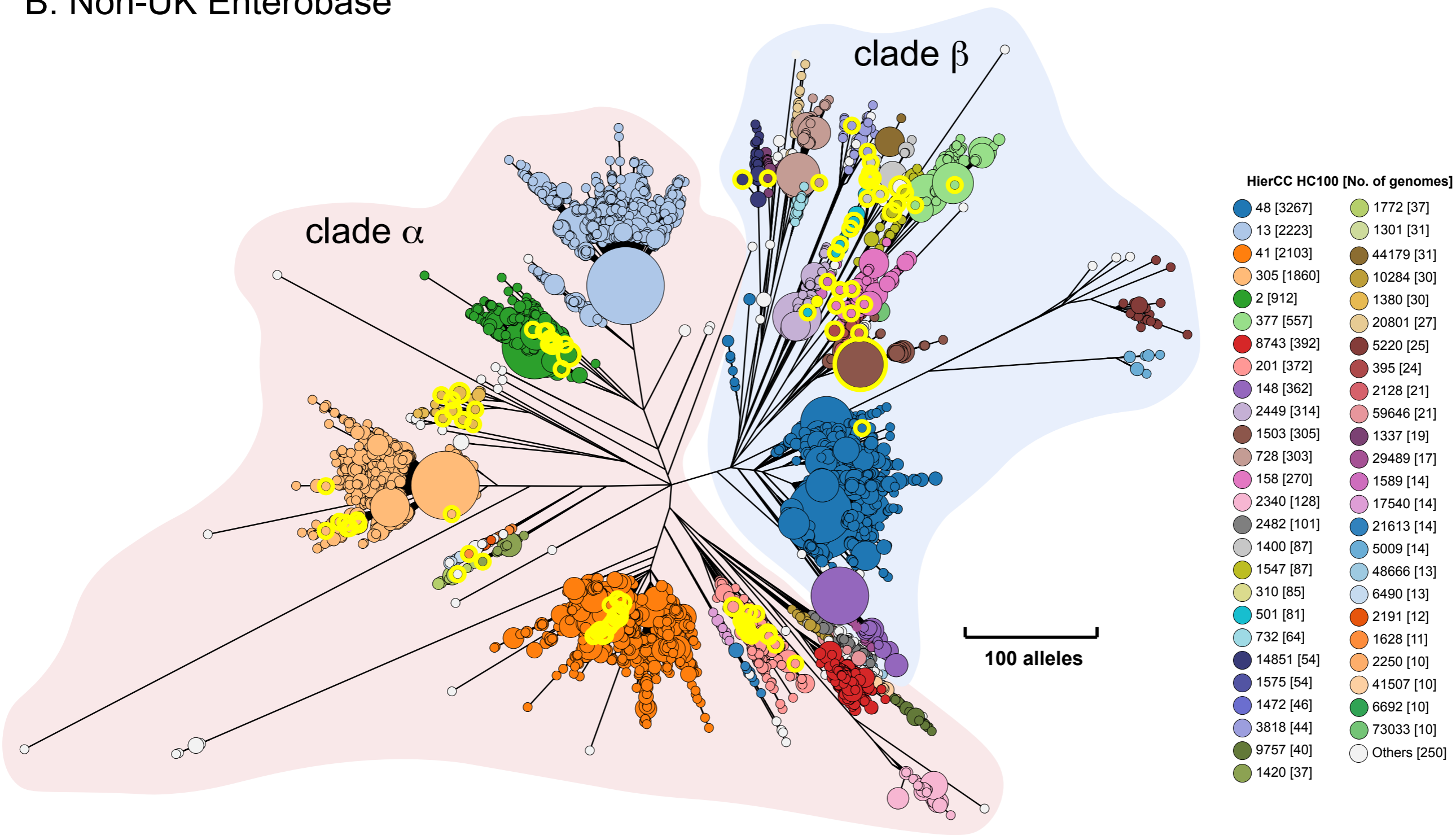

Supplement: S2 Fig — Grapetree visualization based on EnteroBase cgMLST allele profiles, including of (A) 1,693 S. Typhimurium isolates from clinical infections in the UK in 2014 and 2015, and (B) 14,760 genomes selected as the global diversity of S. Typhimurium outside of the United Kingdom in Enterobase database. Nodes are colour coded by EnteroBase HierCC HC100 cluster groups. HierCC groups containing non ST36 isolates in this study yellow circles. Scale indicates number of cgMLST alleles. (PDF) [file pgen.1008850.s002.pdf]

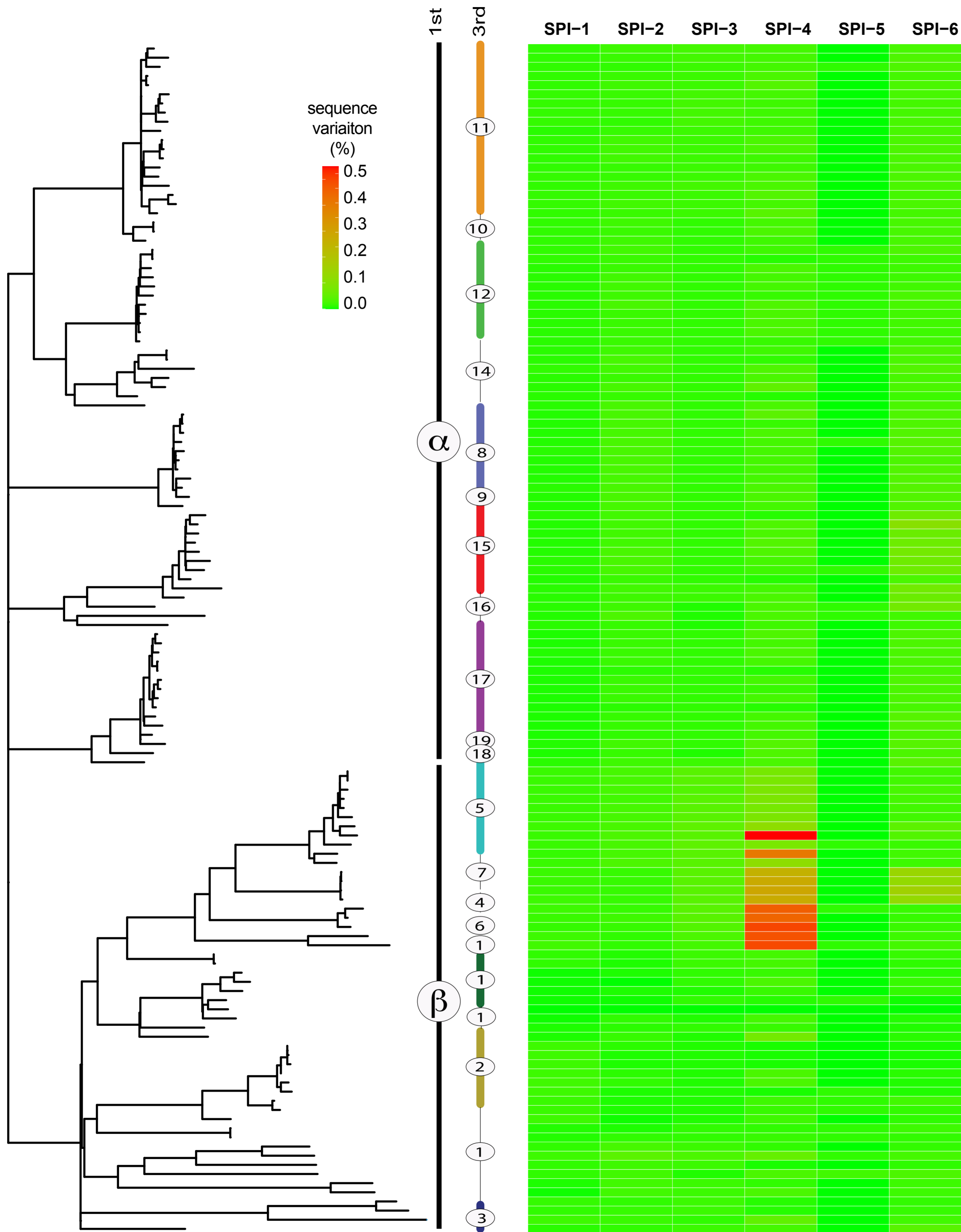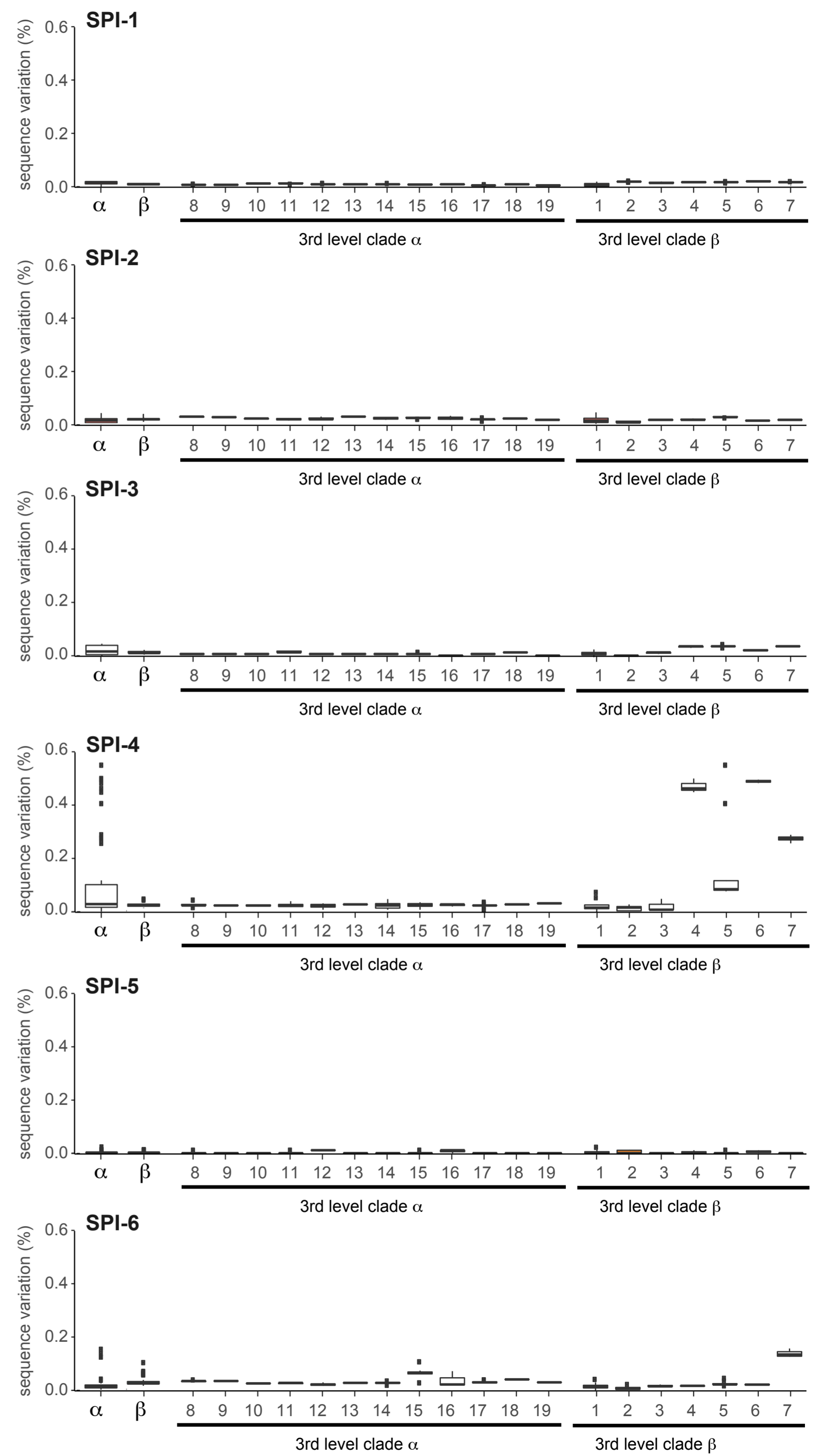

Supplement: S3 Fig — The percent sequence variation including SNPs and deletions in SPI-1, SPI-2, SPI-3, SPI-4, SPI-5 and SPI-6 are indicated from 0% (green) to 0.5% (red). (PDF) [file pgen.1008850.s003.pdf]

S4 Fig

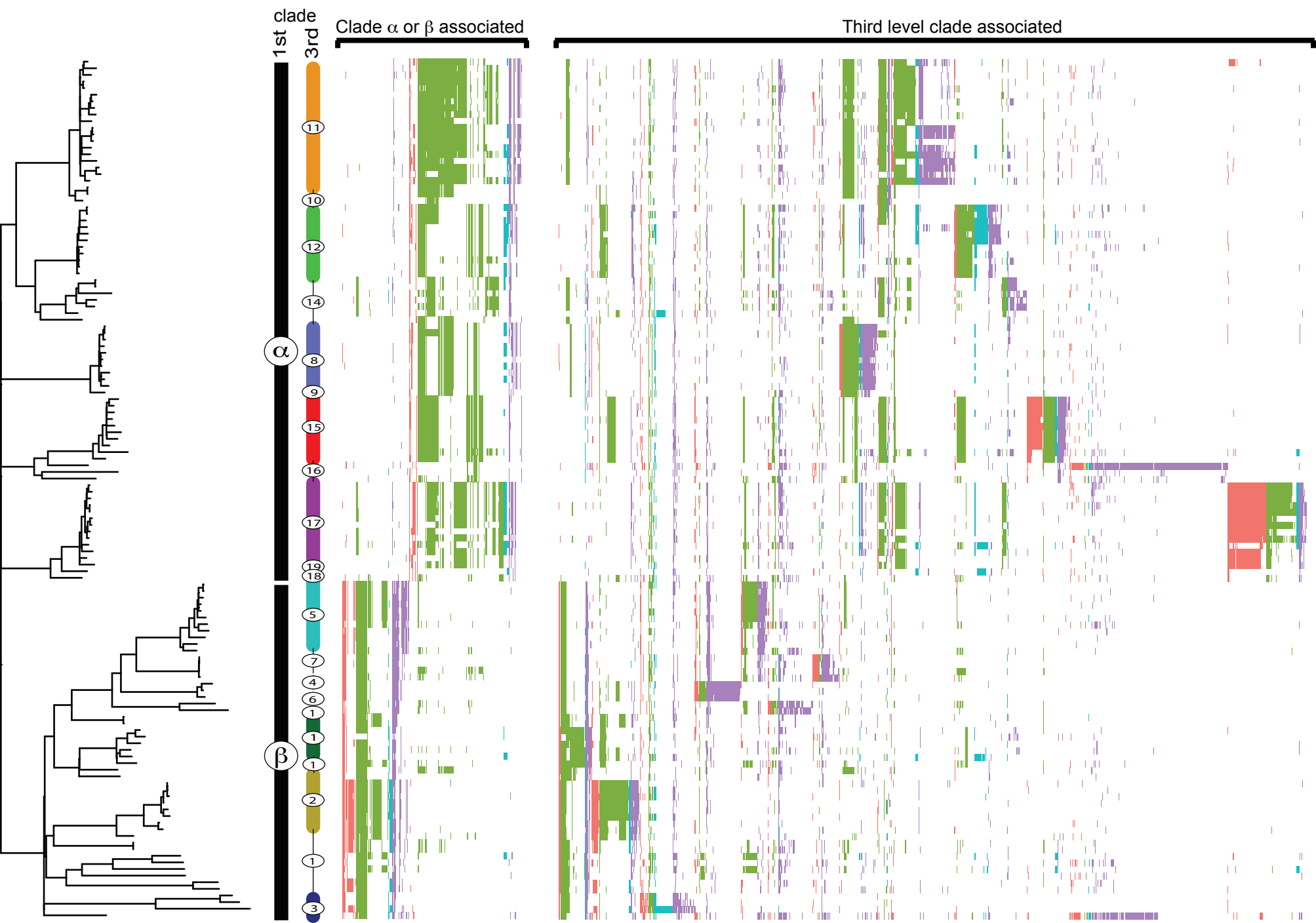

Supplement: S4 Fig — Gene families with a strong clade association in clade α or β (A), or in one of the third level clades (B). Maximum likelihood phylogenetic tree and based on sequence variation (SNPs) in the core genome with reference to S. Typhimurium strain SL1344 (left). Third-level clades are indicated and colour coordinated with that in Fig 1. Genes in each clade were assigned a score based on the number of strains containing the gene within the clade. This score was also calculated for the strains outside the clade. Clade associated genes were defined as genes that had scores greater than the mean plus two SD of the score for all other clades. Genes are colour coded based assignment to non-prophage chromosomal (red), prophage (green), plasmid (blue), or undefined (grey). (PDF) [file pgen.1008850.s004.pdf]

S5 Fig

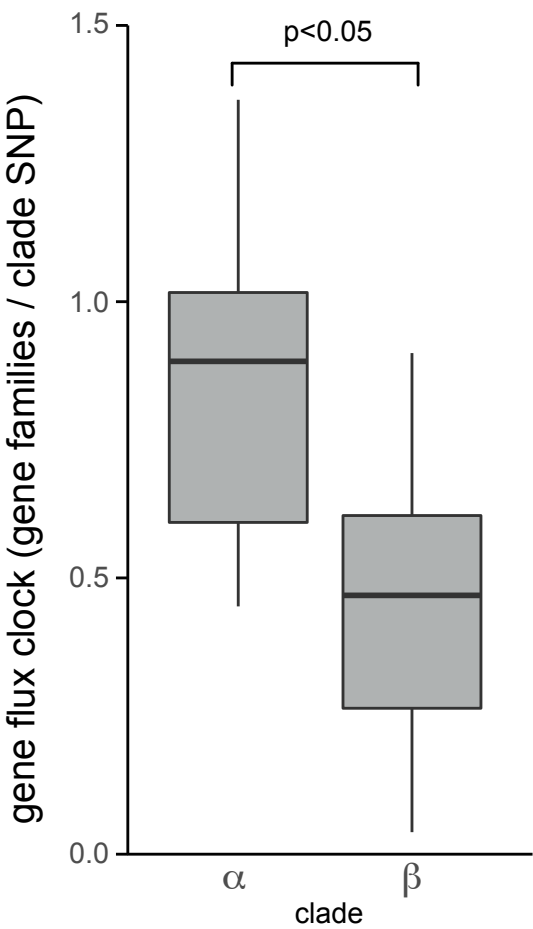

Supplement: S5 Fig — (PDF) [file pgen.1008850.s005.pdf]
