# Supplementary material for: Evolution of Salmonella enterica serotype Typhimurium driven by anthropogenic selection and niche adaptation
Source: PLoS Genet. 2020 Jun 8;16(6):e1008850. doi: 10.1371/journal.pgen.1008850 (PMC7302871; doi:10.1371/journal.pgen.1008850)
Supplement: S6 Table — (DOCX) [file pgen.1008850.s011.docx]

| **Species** | **PHASTER designation** | **PHASTER Accession** | **Cargo Genes** | **Genus** | **Ref** |
| --- | --- | --- | --- | --- | --- |
| “Salmonella virus SPN9CC“ | Salmon_SPN9CC | NC_017985 [1] | None identified | *Lederbergvirus* | [1] |
| “Salmonella virus Gifsy2A and B” | Gifsy2 | NC_010393 [2] | *sseI[3] sodC1[4] grvA[5] gtgA/pipA[6]^*^ gtgE[7] clpP1[8, 9]* | *“Secundusgifsyvirus”* | [2] |
| “Salmonella virus Fels1A and B” | Salmon_Fels_1 | NC_010391 [2] | *pcoC-yebZ[10] yedA[11]* | *“Felsunavirus”* | [2] |
| “Salmonella virus Sal3” | Salmon_118970_sal3 | NC_031940 [12] | *cspJ[13] clpX[14] hcpC[15] mtfA[16] sspH2[17]*  *oatA*  *oafA[18]* | *“Salvirus”* | [12] |
| “Salmonella virus mTmHP1”  “Salmonella virus TmHP1” | Haemop_HP1 | NC_001697 [19]20] | None identified | *Hpunavirus* |  |
| “Salmonella virus Gifsy1A, B, C, D, E, F, G and H” | Gifsy1 | NC_010392 [2] | *pipA/gogA[6, 7] sspH2*  (*artA-artB[20]*) *gogB[21] gipA[22]* | *“Primisgifsyvirus”* | [2] |
| “Salmonella virus BcepMuX” | Burkho_BcepMu | NC_005882 [23] | *rdgB[24]* | *“Saltymuvirus”* |  |
| “Salmonella virus TmST104” | Entero_ST104 | NC_005841 [25] | *pipB2[26]* | *Lederbergvirus* |  |
| “Salmonella virus TmEGF” | Edward_GF_2 | NC_026611 [27] | *mltD[28]* | *Popoffvirus* |  |
| “Salmonella virus TmR2D2” | Entero_mEp237 | NC_019704 [29] | None identified | *“Overgivenvirus”* |  |
| “Salmonella virus ST64BA, B, C, D and E” | Salmon_ST64B | NC_004313 [30] | *cspJ* (*ssek3[31]**) *hcpC*  *mtfA*  (*clpX**) | *“Virulovirus”* |  |
| “Salmonella virus mTmV” | Entero_SfV | NC_003444 [32] | *flxA[33] sopE[34] hin[35]* | *“Salvadorvirus”* |  |
| “Salmonella virus mTmII” | Salmon_SJ46 | NC_031129 [36] | *clpX*  *yafO[37] prtN[38] hin[35]* | *“Salvadorvirus”* |  |
| Salmonella virus BTP1 | Salmon_ST64T | NC_004348 [39] | None identified | *Lederbergvirus* | [40] |
| Salmonella virus BTP5  “Salmonella virus TmB186” | Entero_186 | NC_001317 [41] | None identified | *Eganvirus* | [40] |
| “Salmonella virus TmSEN34” | Salmon_SEN34 | NC_028699 [42] | *pipA/gogA sspH2*  *mltD* | *“Senifvirus”* |  |
| Salmonella virus RE2010 | Salmon_RE_2010 | NC_019488 [43] | None identified | *Felsduovirus* |  |
| “Salmonella virus TmC3PO” | Escher_pro483 | NC_028943 | None identified | *Peduovirus* |  |
| “Salmonella virus TmSEN1” | Salmon_SEN1 | NC_029003 [42] | None identified | *“Carissavirus”* |  |
| “Salmonella virus TmP4” | Entero_P4 | NC_001609 [44] | None identified | *“Boreavirus”* |  |
| “Salmonella virus SopE” | Salmon_Fels_2 | NC_010463 [2] | *sopE* | *“Plomovirus”* |  |

The Species and Genera names have been determined based on the current criteria used by the Bacterial and Archaeal Viruses Subcommittee of the International Committee on Taxonomy of Viruses (ICTV). Proposed and as yet unratified names designated in this work are indicated by being inside quotes (“”). The closest relative proposed by PHASTER software is indicated along with the accession number.

* *sseK3* was only present in the ST64B variant of clade β isolates, and *clpX* only in the variant in clade α isolates

1. Shin, H., et al., *Genomic investigation of lysogen formation and host lysis systems of the Salmonella temperate bacteriophage SPN9CC.* Appl Environ Microbiol, 2014. **80**(1): p. 374-84.

2. McClelland, M., et al., *Complete genome sequence of Salmonella enterica serovar Typhimurium LT2.* Nature, 2001. **413**: p. 852.

3. McLaughlin, L.M., et al., *The Salmonella SPI2 Effector SseI Mediates Long-Term Systemic Infection by Modulating Host Cell Migration.* PLOS Pathogens, 2009. **5**(11): p. e1000671.

4. Sansone, A., et al., *The role of two periplasmic copper- and zinc-cofactored superoxide dismutases in the virulence of Salmonella choleraesuis.* Microbiology, 2002. **148**(Pt 3): p. 719-26.

5. Ho, T.D. and J.M. Slauch, *Characterization of grvA, an antivirulence gene on the gifsy-2 phage in Salmonella enterica serovar typhimurium.* J Bacteriol, 2001. **183**(2): p. 611-20.

6. Jennings, E., et al., *Structure–function analyses of the bacterial zinc metalloprotease effector protein GtgA uncovers key residues required for deactivating NF-κB.* Journal of Biological Chemistry, 2018.

7. Ho, T.D., et al., *Identification of GtgE, a novel virulence factor encoded on the Gifsy-2 bacteriophage of Salmonella enterica serovar Typhimurium.* Journal of bacteriology, 2002. **184**(19): p. 5234-5239.

8. Yamamoto, T., et al., *Disruption of the genes for ClpXP protease in Salmonella enterica serovar Typhimurium results in persistent infection in mice, and development of persistence requires endogenous gamma interferon and tumor necrosis factor alpha.* Infection and immunity, 2001. **69**(5): p. 3164-3174.

9. Knudsen, G.M., et al., *ClpP deletion causes attenuation of Salmonella Typhimurium virulence through mis-regulation of RpoS and indirect control of CsrA and the SPI genes.* Microbiology, 2013. **159**(Pt 7): p. 1497-509.

10. Rensing, C. and G. Grass, *Escherichia coli mechanisms of copper homeostasis in a changing environment.* FEMS Microbiol Rev, 2003. **27**(2-3): p. 197-213.

11. Herrero, A., et al., *Characterization of pUO-StVR2, a virulence-resistance plasmid evolved from the pSLT virulence plasmid of Salmonella enterica serovar Typhimurium.* Antimicrobial agents and chemotherapy, 2008. **52**(12): p. 4514-4517.

12. Paradiso, R., et al., *Complete Genome Sequences of Three Siphoviridae Bacteriophages Infecting Salmonella enterica Serovar Enteritidis.* Genome announcements, 2016. **4**(6): p. e00939-16.

13. Nickerson, K.P., et al., *Salmonella Typhi Colonization Provokes Extensive Transcriptional Changes Aimed at Evading Host Mucosal Immune Defense During Early Infection of Human Intestinal Tissue.* EBioMedicine, 2018. **31**: p. 92-109.

14. Cummings, L.A., et al., *In vivo, fliC expression by Salmonella enterica serovar Typhimurium is heterogeneous, regulated by ClpX, and anatomically restricted.* Molecular Microbiology, 2006. **61**(3): p. 795-809.

15. Mittl, P.R. and W. Schneider-Brachert, *Sel1-like repeat proteins in signal transduction.* Cell Signal, 2007. **19**(1): p. 20-31.

16. Smith, T.D. and A.M. Calvo, *The mtfA transcription factor gene controls morphogenesis, gliotoxin production, and virulence in the opportunistic human pathogen Aspergillus fumigatus.* Eukaryot Cell, 2014. **13**(6): p. 766-75.

17. Quezada, C.M., et al., *A family of Salmonella virulence factors functions as a distinct class of autoregulated E3 ubiquitin ligases.* Proceedings of the National Academy of Sciences of the United States of America, 2009. **106**(12): p. 4864-4869.

18. Hauser, E., et al., *Different mutations in the oafA gene lead to loss of O5-antigen expression in Salmonella enterica serovar Typhimurium.* Journal of Applied Microbiology, 2010. **110**(1): p. 248-253.

19. Esposito, D., et al., *The complete nucleotide sequence of bacteriophage HP1 DNA.* Nucleic Acids Res, 1996. **24**(12): p. 2360-8.

20. Uchida, I., et al., *Salmonella enterica serotype Typhimurium DT104 ArtA-dependent modification of pertussis toxin-sensitive G proteins in the presence of [32P]NAD.* Microbiology, 2009. **155**(Pt 11): p. 3710-8.

21. Coombes, B.K., et al., *Genetic and molecular analysis of GogB, a phage-encoded type III-secreted substrate in Salmonella enterica serovar typhimurium with autonomous expression from its associated phage.* J Mol Biol, 2005. **348**(4): p. 817-30.

22. Stanley, T.L., C.D. Ellermeier, and J.M. Slauch, *Tissue-specific gene expression identifies a gene in the lysogenic phage Gifsy-1 that affects Salmonella enterica serovar typhimurium survival in Peyer's patches.* J Bacteriol, 2000. **182**(16): p. 4406-13.

23. Summer, E.J., et al., *Burkholderia cenocepacia phage BcepMu and a family of Mu-like phages encoding potential pathogenesis factors.* J Mol Biol, 2004. **340**(1): p. 49-65.

24. Bradshaw, J.S. and A. Kuzminov, *RdgB acts to avoid chromosome fragmentation in Escherichia coli.* Molecular Microbiology, 2003. **48**(6): p. 1711-1725.

25. Tanaka, K., et al., *Molecular characterization of a prophage of Salmonella enterica serotype Typhimurium DT104.* J Clin Microbiol, 2004. **42**(4): p. 1807-12.

26. Henry, T., et al., *The Salmonella effector protein PipB2 is a linker for kinesin-1.* Proceedings of the National Academy of Sciences, 2006. **103**(36): p. 13497.

27. Yasuike, M., et al., *Full-genome sequence of a novel myovirus, GF-2, infecting Edwardsiella tarda: comparison with other Edwardsiella myoviral genomes.* Arch Virol, 2015. **160**(8): p. 2129-33.

28. Xu, Z., et al., *Mutation of a novel virulence-related gene mltD in Vibrio anguillarum enhances lethality in zebra fish.* Research in Microbiology, 2011. **162**(2): p. 144-150.

29. Uc-Mass, A., et al., *An orthologue of the cor gene is involved in the exclusion of temperate lambdoid phages. Evidence that Cor inactivates FhuA receptor functions.* Virology, 2004. **329**(2): p. 425-433.

30. Mmolawa, P.T., H. Schmieger, and M.W. Heuzenroeder, *Bacteriophage ST64B, a genetic mosaic of genes from diverse sources isolated from Salmonella enterica serovar typhimurium DT 64.* J Bacteriol, 2003. **185**(21): p. 6481-5.

31. Brown, N.F., et al., *Salmonella Phage ST64B Encodes a Member of the SseK/NleB Effector Family.* PLOS ONE, 2011. **6**(3): p. e17824.

32. Allison, G.E., et al., *Complete genomic sequence of SfV, a serotype-converting temperate bacteriophage of Shigella flexneri.* J Bacteriol, 2002. **184**(7): p. 1974-87.

33. Ide, N. and K. Kutsukake, *Identification of a novel Escherichia coli gene whose expression is dependent on the flagellum-specific sigma factor, FliA, but dispensable for motility development.* Gene, 1997. **199**(1): p. 19-23.

34. Mirold, S., et al., *Isolation of a temperate bacteriophage encoding the type III effector protein SopE from an epidemic &lt;em&gt;Salmonella typhimurium&lt;/em&gt; strain.* Proceedings of the National Academy of Sciences, 1999. **96**(17): p. 9845.

35. Hughes, K.T., P. Youderian, and M.I. Simon, *Phase variation in Salmonella: analysis of Hin recombinase and hix recombination site interaction in vivo.* Genes & Development, 1988. **2**(8): p. 937-948.

36. Yang, L., et al., *Characterization of a P1-like bacteriophage carrying CTX-M-27 in Salmonella spp. resistant to third generation cephalosporins isolated from pork in China.* Scientific Reports, 2017. **7**: p. 40710.

37. Brown, J.M. and K.J. Shaw, *A Novel Family of &lt;em&gt;Escherichia coli&lt;/em&gt; Toxin-Antitoxin Gene Pairs.* Journal of Bacteriology, 2003. **185**(22): p. 6600.

38. Matsui, H., et al., *Regulation of pyocin genes in Pseudomonas aeruginosa by positive (prtN) and negative (prtR) regulatory genes.* J Bacteriol, 1993. **175**(5): p. 1257-63.

39. Mmolawa, P.T., et al., *Genomic structure of the Salmonella enterica serovar Typhimurium DT 64 bacteriophage ST64T: evidence for modular genetic architecture.* J Bacteriol, 2003. **185**(11): p. 3473-5.

40. Abraham, S., et al., *Isolation and plasmid characterization of carbapenemase (IMP-4) producing Salmonella enterica Typhimurium from cats.* Sci Rep, 2016. **6**: p. 35527.

41. Portelli, R., et al., *The late-expressed region of the temperate coliphage 186 genome.* Virology, 1998. **248**(1): p. 117-30.

42. Mikalová, L., et al., *Novel Temperate Phages of Salmonella enterica subsp. salamae and subsp. diarizonae and Their Activity against Pathogenic S. enterica subsp. enterica Isolates.* PLOS ONE, 2017. **12**(1): p. e0170734.

43. Hanna, L.F., et al., *Characterization of the ELPhiS prophage from Salmonella enterica serovar Enteritidis strain LK5.* Appl Environ Microbiol, 2012. **78**(6): p. 1785-93.

44. Halling, C., et al., *DNA sequence of satellite bacteriophage P4.* Nucleic Acids Res, 1990. **18**(6): p. 1649.
